# RNA quality and protamine gene expression after storage of mouse testes under different conditions

**DOI:** 10.1371/journal.pone.0314013

**Published:** 2024-11-21

**Authors:** Nerea Latorre, Beatriz A. Dorda, Isabel Rey, Eduardo R. S. Roldan, Ana Sanchez-Rodriguez

**Affiliations:** 1 Department of Biodiversity and Evolutionary Biology, Museo Nacional de Ciencias Naturales (CSIC), Madrid, Spain; 2 Tissues and DNA Collection, Museo Nacional de Ciencias Naturales (CSIC), Madrid, Spain; Chung-Ang University College of Engineering, REPUBLIC OF KOREA

## Abstract

Protamines are proteins responsible for condensing sperm chromatin. There are two protamines whose ratio remains constant in each species and which is related to fertility. To quantify their expression, it is necessary to have a good protocol of sample collection (*i*.*e*., RNA stabilizing buffers and temperature conditions). The aim of this work was to compare gene expression of protamines, with analysis of RNA quality and ratios, in testis samples from wild-derived mice, *Mus musculus*, preserved in different buffers (RNAlater^®^ or Nucleic Acid Preservation–NAP–buffer) and different temperatures (room temperature -RT-, 4°C, -20°C, -80°C or liquid nitrogen) for different times (one week, one month, 3 months and one year). The relative abundance of protamine expression was assessed by qPCR using *18S rRNA* as housekeeping. The results showed that the preservation of testes in RNAlater^®^ or NAP buffer at -80°C afforded equivalent good preservation as in somatic tissues. Testis samples stored at RT in both buffers for 1 week resulted in a similar RNA quality and protamine expression over time. Moreover, samples in RNAlater^®^ stored at RT, 4°C, -20°C and -80°C, were analyzed after 24 h, 7 days, 30 days, 90 days or 365 days; samples stored at RT resulted in a loss of RNA quality but protamine ratio was maintained up to 90 days. Samples stored at 4°C and -20°C showed similar values of RNA integrity and protamine expression than those stored at -80°C. Finally, we stored testis samples at -80°C or -196°C, after initial snap-freezing in liquid nitrogen. Both methods afforded very good preservation of RNA integrity and protamine expression. These results open new possibilities for the collection, transport and storage of testes samples under field conditions.

## Introduction

The formation of male gametes takes place during spermatogenesis. Spermiogenesis is the final phase of this process and involves differentiation of spermatids into spermatozoa. The spermatids undergo major structural and molecular changes, such as elongation of the nucleus and condensation of the chromatin, and each spermatozoon acquires the shape and dimensions characteristic of its species [1]. DNA hypercondensation is of vital importance for inhibiting DNA transcription and protecting the genome from environmental damage. It is achieved by protamines 1 and 2 (PRM1 and PRM2), which are positively charged, and that replace histones during sperm differentiation. The protein PRM1 is present in all mammals [2], whereas PRM2 has been described in some species such as primates, rodents, equids, bovids, and a subset of eutherian mammals [3]. It is essential that the protamine ratio (PRM1/PRM2) is maintained for normal sperm function. Moreover, numerous studies have shown the relationship between the ratio of both protamines and the competitive ability of spermatozoa [2], since differences in genes encoding both protamines and their promoters directly affect spermatozoa morphology, in particular the shape and size of the sperm head [4]. In mammals, sperm head shape and size can influence cell hydrodynamics and swimming speed. Spermatozoa having elongated and smaller heads, relative to flagellum length, have a higher swimming velocity [5, 6]. Therefore, changes in the regulatory regions of the *Prm1* and *Prm2* genes could increase the efficiency of DNA condensation in the sperm head and thus affect sperm performance [7]. Since protamines are present in all mammals, it is important to understand the impact of changes in these proteins on sperm differentiation, as well as on the transmission of genetic material.

When studying the evolution of protamine genes, it is necessary to analyze the diversity of gene expression in wild species. Sampling in the field has a limitation associated with the difficulty of preserving tissue samples in suitable conditions until they reach the laboratory. Once collected, the samples must be properly preserved to obtain RNA with adequate integrity and quality to obtain complementary DNA (cDNA) from which the expression of protamines can be quantified. DNA and RNA undergo degradation with time and temperature, being faster in the case of RNA [8]. Stabilizing buffers, such as RNAlater^®^, can preserve RNA for 4 weeks at 2 to 8°C, 1 week at 15–25°C, and up to 1 day at 37°C [9]. However, these buffers are expensive and sometimes field work requires more time and is carried out at suboptimal conditions. To overcome these limitations, a laboratory-made buffer has been developed [9], named Nucleic Acid Preservation (NAP) buffer. This buffer is able to preserve RNA quality and quantity of somatic tissues such as liver, brain, muscle, ear or tail for 7–8 weeks and blood for 10 months at room temperature.

Besides the use of a suitable RNA stabilizing buffer, it is also important to determine the optimal storage temperature for samples. Some studies have reported that cryopreservation is the best way to preserve RNA integrity [10]. This is executed by snap-freezing samples in dry ice or liquid nitrogen (LN_2_), which allows samples to be flash-frozen at temperatures close to -200°C and then stored at -80°C. This method provides high sample integrity and also eliminates contamination risks [10]. However, frozen tissue is more difficult to handle and homogenize than non-frozen samples, and the thawing process may result in partial degradation of RNA [11]. In addition, the use of LN_2_ is not always possible in the field or in laboratories. Therefore, several studies have considered the option of preserving samples by immersing them in RNAlater^®^ followed by freezing at -80°C, or snap-freezing them (tumor tissue [12]; liver [13]; somatic tissues [14]), having good results of RNA quality. The specific mechanisms by which these buffers preserve genetic material is not known, but Kilpatrick *et al*. [15] suggest that the presence of EDTA might protect the DNA during the extraction of DNA process.

The aim of the present study was to examine different methods of storage and preservation of testis samples for the study of RNA quality and protamine gene expression. For this purpose, first, we examined the possibility of testes preservation at -80°C in NAP buffer and RNAlater^®^ comparing it with the preservation of liver, as this somatic tissue has been reported to be well preserved in NAP buffer [9]. Subsequently, both buffers were tested in the conservation of testes at room temperature for 7 days, simulating field conditions where it is not possible to perform cryopreservation of samples. We also evaluated the effect of different temperatures (22°C -room temperature-, 4°C, -20°C and -80°C) in samples kept in RNAlater^®^ for one year, as well as the effect of storing samples at -80°C or in LN_2_ after snap-freezing. These conditions were evaluated after different times of storage (24 h–time 0 –, 7 days, 30 days, 90 days and 365 days, depending on the conditions).

## Methods

### Animals and tissue collection

All animal procedures and handling followed the Spanish Animal Protection Regulation RD53/2013, the European Union Regulation 2010/63, the ARRIVE guidelines, and had the approval of CSIC’s ethics committee and the Comunidad de Madrid (28079-47-A).

A total of 24 males of *Mus musculus musculus* were used in this study. These animals are derived from wild specimens that have been bred in captivity for several generations at the University of Montpellier, France. Individuals were reared and cared for in the Animal Facility of the National Museum of Natural Sciences (CSIC) until they reached sexual maturity at 60 days of age.

Mice were sacrificed by cervical dislocation. Testes and liver were collected to compare the effect of NAP buffer and RNAlater^®^ under different conditions of storage. Each testis was cut into three sections of similar size, without removing the tunica albuginea, since it is reported that the presence of the tunica (pricked or when testes are cut) has a good impact in cell viability after thawing [16]. Each sample was randomly assigned to the different preservation conditions.

RNAlater^®^ was purchased from Thermo Fisher Scientific (Cat. # AM7020, Waltham, MA, USA) and NAP buffer was prepared with the following composition [9]: ethylenediamine tetraacetic acid (EDTA) dihydrate 0.019 M disodium salt (Cat. # E9884, Merk/MilliporeSigma, St. Louis, MA, USA), sodium citrate dihydrate 0.018 M trisodium salt (Cat. # 6132-04-3, St. Louis, MA, USA, Merk/MilliporeSigma), ammonium sulphate 3.8 M (Cat. # 204501, Merk/MilliporeSigma) and adjusted to pH 5.2.

### Experimental design

**Fig 1:**
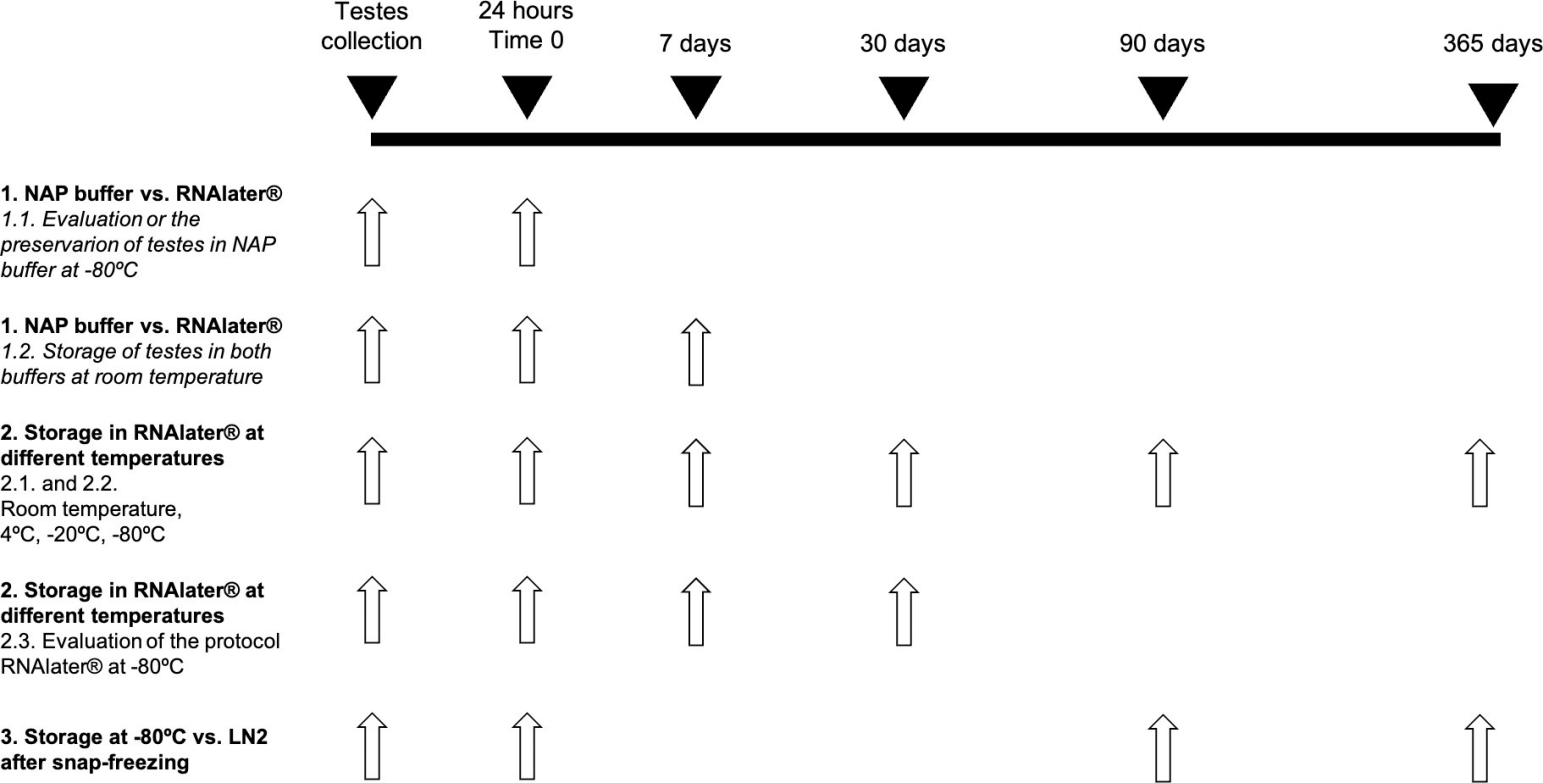
Flowchart of the experimental design. Time line represents the days when samples were analyzed for RNA purity and integrity and protamine expression. White arrow indicates the time points when samples were assessed in each experiment. On the left side, the experiment performed is indicated, following the numbering given in the Results section.

The experimental design is depicted in Fig 1. In the first experiment we evaluated the effect of RNAlater^®^ and NAP buffer in liver and testes stored at -80°C for 24 h to validate the use of NAP buffer in testes. The second experiment was performed with testes in order to assess the impact of both buffers during storage at room temperature for 7 days, carrying out evaluations at time 0 (24 h after collection) and after 7 days from collection. The third experiment was designed to examine samples stored in RNAlater^®^, at different temperatures (room temperature, 4°C, -20°C or -80°C) for different times (0, 7, 30, 90 and 365 days). In this experiment, RNAlater^®^ was used following the manufacturer’s instructions (*i*.*e*., leaving samples overnight at 4°C, then discarding the RNAlater^®^ for samples to be stored at -20°C or -80°C); to analyze the effect of RNAlater^®^ in frozen samples, we also compared tissues kept for 30 days at -80°C both without and with buffer. The last experiment examined differences between testes samples stored at -80°C or LN_2_ after snap-freezing for periods of 90 and 365 days.

### RNA extraction

For RNA extraction, the E.Z.N.A.^®^ HP Total RNA extraction kit (Cat. # R6812-01, Omega-Biotek, Norcross, GA, USA) was used following the manufacturer’s instructions. Briefly, each testis piece was homogenized with 700 μl of lysis buffer. After centrifugation for 5 min at 13,400 rpm, 70% ethanol was added to the supernatant and mixed by pipetting. The liquid was then transferred to an RNA spin column and centrifuged; the resulting liquid was discarded. After several washes and centrifugations of the column, a final centrifugation under vacuum was performed to dry the membrane. A volume of 50 μl of Ambion^TM^ DEPC-Treated water (Cat. # AM9919, Invitrogen, Life Technologies, Waltham, MA, USA) was added to elute the resulting RNA from the column. RNA samples were preserved on ice for immediate measurement of RNA concentration and integrity.

### RNA concentration and integrity

The RNA concentration of the samples was measured with a spectrophotometer (NanoDrop 2000, Walthman, MA, USA, Thermo Fisher Scientific), which provides absorbance data at different wavelengths (230, 260 and 280 nm), as well as the relationships between them. To consider RNA to be of maximum purity, the sample at A260/A280 should have a value between 2 and 2.2.

The ratio of the ribosomal bands (28S/18S) has conventionally been studied as the primary indicator of RNA integrity, with a ratio of 2.0 considered to be typical of ‘high quality’ intact RNA [17, 18]. Therefore, 1.8% agarose gel electrophoresis was performed, adding 3.5 μl of SYBR^®^ Safe (Cat. # S33102, Walthman, MA, USA, Thermo Fisher Scientific), to provide highly sensitive staining to visualize nucleic acids. Samples and the molecular weight marker (100 bp ladder, BioTools DNA markers, Madrid, Spain) were loaded, and electrophoresis was run at 80 V for 40 min.

Samples were also analyzed at the Proteomics and Genomics Service of the Centre for Biological Research (CSIC) (Madrid, Spain) to evaluate RNA integrity (RQI, RNA quality indicator), using the Experion^TM^ Automated Electrophoresis System (Bio-Rad, Hercules, CA, USA). A small amount of RNA was separated in the channels of the microchips according to its molecular weight and then detected by laser-induced fluorescence. The result was displayed as an electropherogram where the amount of fluorescence measured correlated with the amount of RNA of a given size. The RQI was estimated in a scale from 1 to 10, 1 being completely disintegrated RNA and 10 being non-degraded RNA. The ratio 28S/18S was also obtained from these analyses; values close to 2 mean good RNA integrity.

### Reverse-transcription of RNA and obtaining cDNA

Reverse-transcription polymerase chain reaction (RT-PCR) was performed on the extracted RNA to obtain cDNA (final volume 50 μl), which was subsequently used as a template in the qPCR reactions. To obtain cDNA, the Superscript III First-Strand Synthesis kit (Cat. # 18080051, Invitrogen^TM^, Thermo Fisher Scientific) was performed according to the manufacturer’s instructions and using OligodT primers for the reverse-transcription. The resulting DNA concentration of each sample was determined by NanoDrop. Samples were stored at -20°C for further analysis.

### Quantitative PCR (qPCR)

The iQ™ SYBR^®^ Green Supermix kit (Cat. # 1708880, Bio-Rad) was used for assessing the relative gene expression of *Prm1* and *Prm2*. Primers previously reported (designed across an exon-exon junction) for *Mus musculus* to analyze expression of *Prm1* (Fw: 5’-AGGCGAAGATGTCGCAGACG-3’; Rv: 3’-CCTTATGGTGTATGAGCGGCGG-5’) and *Prm2* (Fw: 5’-ACAAGAGGCGTCGGTCATGC-3’; Rv: 3’-GTGCCTCCTACATTTCCTGCACC-5’) and of *18S rRNA* housekeeping (Fw: 5’-TGCAATCCCCGATCCCCATCAC-3’; Rv: 3’-AGAGGGACAAGTGGCGTTCAGC-5’) genes were used [4]. The *18S rRNA* gene was used to normalize the fluorescence obtained with the specific gene of each sample, as the gene expression of this gene remains constant regardless of the treatment used. A thermal cycler (QuantStudio™ 3 Real-Time PCR Instrument, 96-well 0.1 ml Block, Applied Biosystems, Waltham, MA, USA) was used with a 95°C cycle for 10 min, followed by 40 cycles of 15 s at 95°C and 1 min at 62°C. A melt-curve was performed at the end of the cycles (95°C for 15 min, 1 h at 50°C and 15 min at 95°C).

The results of each real-time PCR analysis were analyzed as previously described [19]. Non-template controls (NTC) were negative (Cq undetermined). The Thermo Fisher Connect software was used to obtain the Cq values, that define the PCR cycle in which the SYBR^®^ Green fluorescence signal crosses the arbitrarily determined threshold value [19]. The Ct value of the genes of interest was normalized to the Ct of the housekeeping *18S rRNA* on each plate (ΔCt). The *Prm1/Prm2* expression ratio was then calculated.

### Statistical analyses

The statistical package GraphPad Prism 9.0 (GraphPad Software, Boston, MA, USA) was used for statistical analyses. Data (RNA purity and integrity, protamine expression measured by ΔCt and *Prm1/Prm2* expression ratio) were analyzed by two-way ANOVA, with the experimental groups and different times as a fixed effect, and using the Tukey’s multiple comparison test to compare means. Results are shown as means ± standard error of the mean (SEM) and significant differences were considered when p-value < 0.05.

## Results

### 1. Comparison between NAP buffer and RNAlater^®^

#### 1.1. Evaluation of the preservation of testes in NAP buffer at -80°C

To examine the ability of NAP buffer for the storage of testis tissue, we compared this buffer with RNAlater^®^. We also compared testicular with liver tissue, storing them in both buffers. When samples were stored for 24 h at -80°C in both buffers, the values of A260/A280 were > 2 for liver (2.09±0.01 and 2.12±0.01, RNAlater^®^ and NAP buffer, respectively) and testes (2.09±0.002 and 2.1±0.01, respectively, see Table 1 in S1 File for data), indicating that the purity of the RNA extracted from both tissues was optimal. The RQI (10±0, see Table 1 in S1 File for data) and 28S/18S ratio (> 2.4, see Table 1 in S1 File for data) values measured by the Experion^TM^ Automated Electrophoresis System indicated high RNA integrity. No significant differences were observed between tissues nor between the two preservation buffers. To assess RNA integrity, 1.8% agarose electrophoresis gels were run. In both tissues, bands representing the 28S and 18S rRNA subunits were clearly visible, indicating high RNA integrity (S1 and S2 Figs).

#### 1.2. Storage of testes in RNAlater^®^ or NAP buffer at room temperature for 7 days

**Fig 2:**
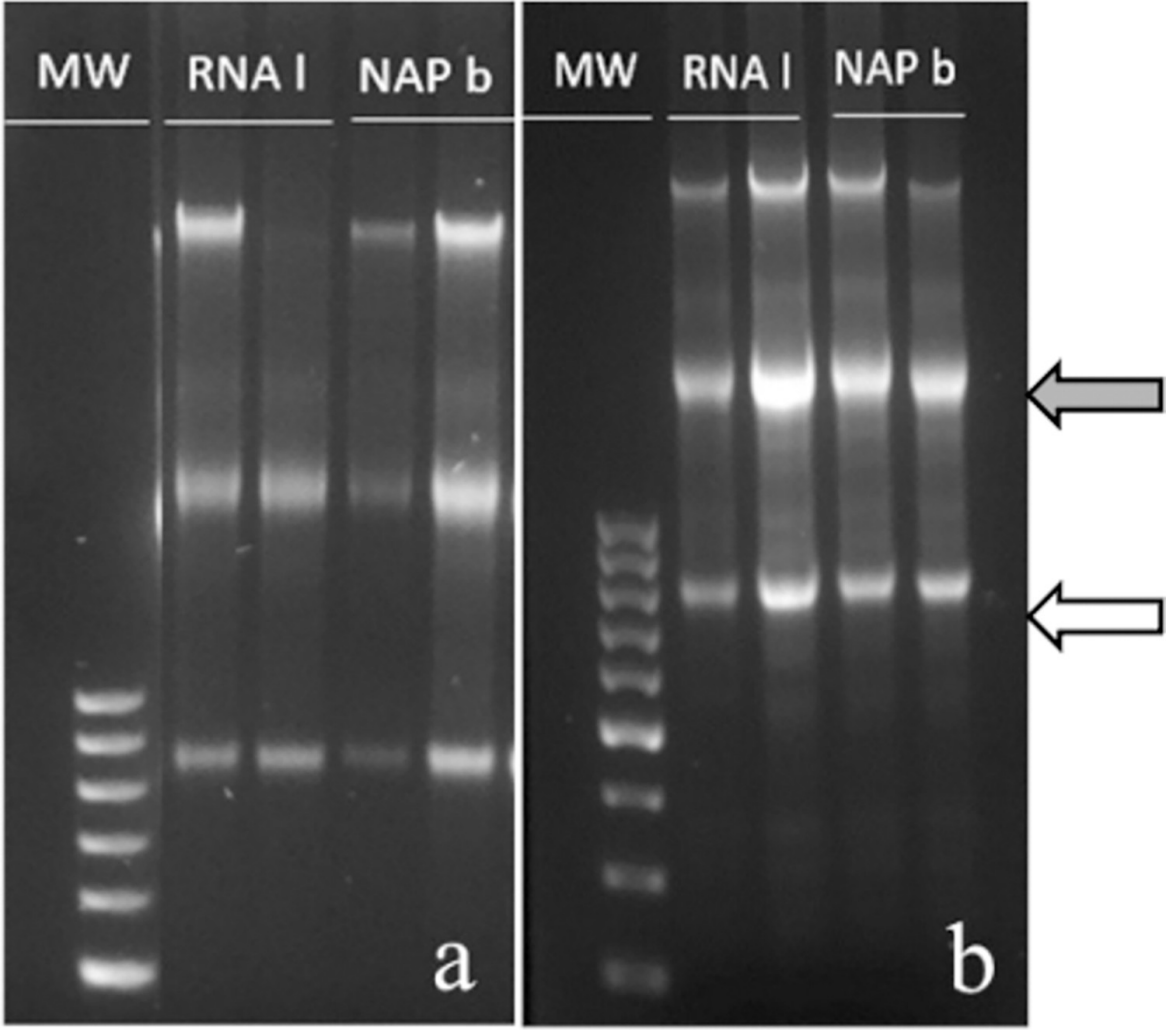
Agarose gel electrophoresis of testis samples stored at room temperature for 7 days. Samples were preserved in RNAlater^®^ or NAP buffer for different times at room temperature (20–22°C). **a**: samples stored for 24 h (time 0); **b**: samples stored for 7 days. MW: molecular weight marker; RNA l: RNAlater^®^; NAP b: NAP buffer. Grey arrow: rRNA28S; white arrow: rRNA18S.

There were no significant differences between RNA concentration in testis samples stored at room temperature (22°C) for up to 7 days. The absorbance ratio A260/280 was > 2 in samples stored for 24 h or 7 days (2.07±0.01 and 2.08±0.01, RNAlater^®^ and NAP buffer, respectively, see Table 2 in S1 File for data). RQI values for samples of times 0 (9.98±0.02 *vs*. 9.97±0.02, for RNAlater^®^ and NAP buffer, respectively, see Table 2 in S1 File for data) and 7 days (9.6±0.14 vs. 9.82±0.16, respectively, see Table 2 in S1 File for data) were similar between buffers tested. 28S/18S ratio was similar when storing testes in RNAlater^®^ and NAP buffer at time 0 (2.21±0.08 vs. 2.31±0.11, respectively, see Table 2 in S1 File for data), whereas it significantly decreased after 7 days of storage in RNAlater^®^ (1.65±0.21, p = 0.0203) but the difference was not significant in NAP buffer (1.92±0.16, p = 0.1068); however, there were no significant differences between both buffers after 7 days of storage. RNA integrity was assessed in 1.8% agarose electrophoresis gels. Images of samples analyzed at time 0 and 7 days showed two clear bands, corresponding to 28S and 18S rRNA subunits (Fig 2). cDNA was obtained for RT-PCR from the RNA.

**Fig 3:**
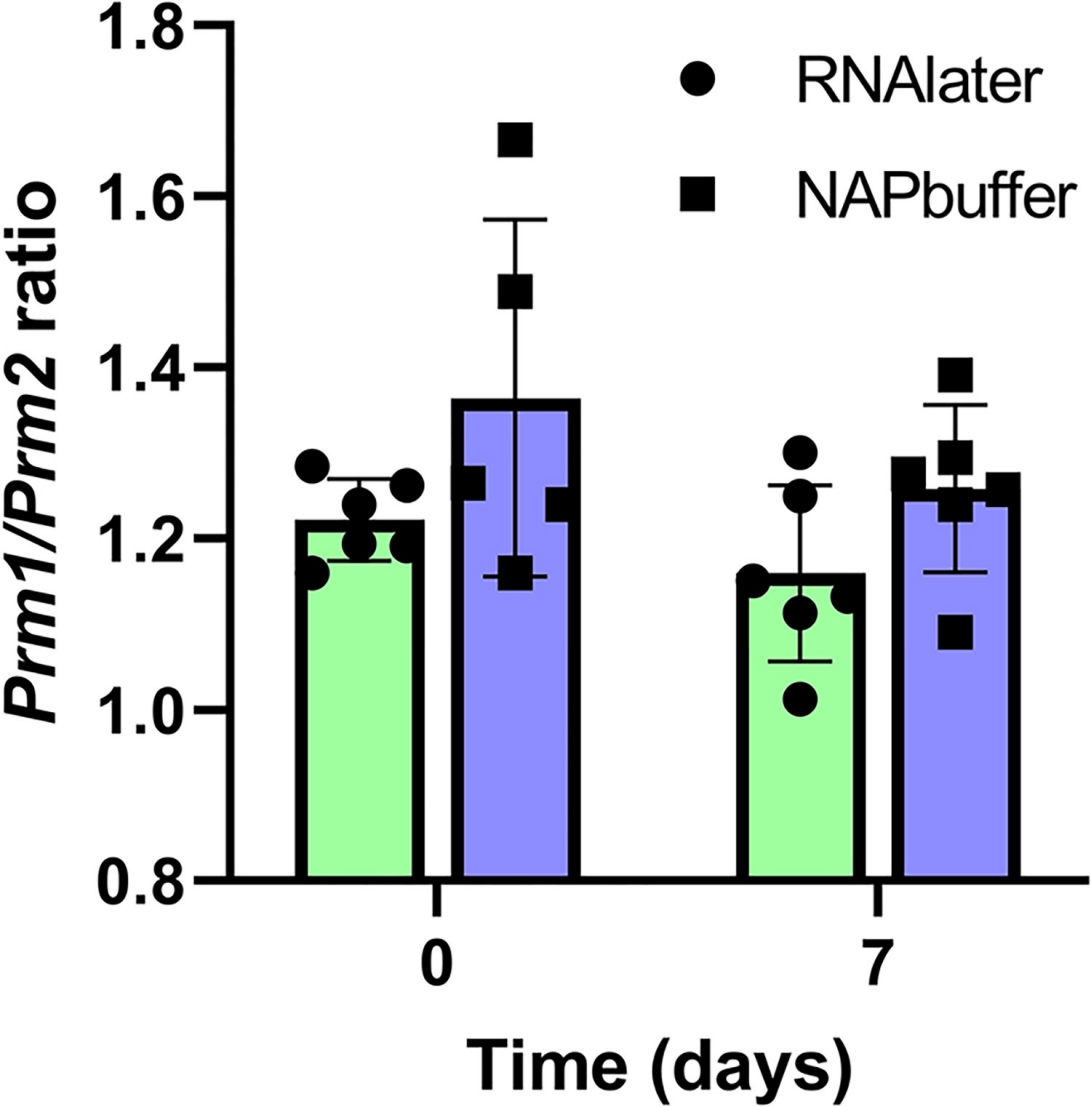
Protamine ratio (*Prm1/Prm2*) in testes samples stored in different preservation buffers for 7 days. Light green: RNAlater^®^; light blue: NAP buffer. Data are means±SEM; individual values are represented (N = 6).

When protamine expression was examined, no significant differences in *Prm1* expression were found between samples stored in RNAlater^®^ and NAP buffer. After 7 days, *Prm1* expression was significantly higher in samples stored in RNAlater^®^ compared to time 0 (6.36±0.63 vs. 3.32±0.57, respectively, p = 0.0093, see Table 3 in S1 File for data). The same occurred for *Prm2* expression–both buffers maintained similar expression in both times, and it significantly increased 7 days after storing samples in RNAlater^®^ (2.71±0.5 vs. 5.15±0.4, respectively, p = 0.0416, see Table 3 in S1 File for data). The *Prm1*/*Prm2* ratio was similar between RNAlater^®^ and NAP buffer in time 0 (1.22±0.02 vs. 1.36±0.09, respectively) and after 7 days of storage (1.16±1.14 vs. 1.26±0.04, respectively) (Fig 3, see Table 4 in S1 File for data).

### 2. Storage of testes in RNAlater^®^ at different temperatures for different times

#### 2.1. RNA quality

As seen in Table 1 (see Table 1 in S2 File for data), all samples stored for 24 h (time 0) showed high RNA purity and integrity, since the A260/A280 ratio value was > 2 and RQI values were close to the highest value (10). After 7, 30, 90 or 365 days of storage, there were no significant differences in both RNA concentration and absorbance values. After 7 days of preservation, RQI was similar between different temperatures, but the storage at room temperature showed significant lower values (p = 0.0189) of the ratio 28S/18S, which were evident also after 365 days (p = 0.0244). The same occurred with the samples stored at 4°C, with significant differences (p = 0.0424) of the ratio 28S/18S between time 0 and 365 days. After one year, samples preserved at -20°C and -80°C had higher but no significant values of RQI than samples at room temperature and the ratio 28S/18S was higher in those temperatures than at room temperature and 4°C.

**Table 1:** RNA quality of testes samples stored in RNA later^®^ at different temperatures for different times.

Temperature	Time (days)	A260/A280	RQI	Ratio 28S/18S
22°C	0 (N = 9)	2.11±0.01	9.98±0.02	2.41±0.1 ^#^ ^β^
22°C	7 (N = 6)	2.32±0.15	9.7±0.07	1.46±0.07 ^#^
22°C	30 (N = 6)	2.11±0.11	5.55±2.85	1.3±0.21
22°C	90 (N = 3)	2.13±0.04	5.33±2.76	1.12±0.33
22°C	365 (N = 3)	2.03±0.04	4.03±2.46	0.57±0.24 ^β^
4°C	0 (N = 9)	2.14±0.02	9.97±0.03	2.55±0.1 ^†^
4°C	7 (N = 6)	2.15±0.02	9.92±0.05	2.37±0.08
4°C	30 (N = 6)	1.9±0.03	8.63±0.56	2.6±0.65
4°C	90 (N = 3)	2.11±0.02	9.33±0.62	2.24±0.27
4°C	365 (N = 3)	2.07±0.01	9.43±0.17	1.24±0.11 ^†^
-20°C	0 (N = 9)	2.11±0.02	10±0	2.49±0.1
-20°C	7 (N = 6)	2.53±0.35	9.7±0.11	2.12±0.19
-20°C	30 (N = 6)	1.94±0.14	8.78±0.84	3.93±0.58
-20°C	90 (N = 3)	2.09±0.01	10±0	2.27±0.16
-20°C	365 (N = 3)	2.07±0.02	9.97±0.03	1.93±0.14
-80°C	0 (N = 9)	2.13±0.01	10±0	2.55±0.1
-80°C	7 (N = 6)	2.13±0.01	9.8±0	2.2±0.08
-80°C	30 (N = 6)	2.03±0.04	7.87±0.9	4.74±1.03
-80°C	90 (N = 3)	2.11±0.01	9.3±0.7	2.14±0.1
-80°C	365 (N = 3)	2.04±0.02	10±0	1.92±0.15

Samples were preserved in RNA later^®^ at different temperatures for up to 365 days. Data are means±SEM.

^#^ Significant differences between time 0 and 7 at 22°C (p = 0.0189).

^β^ Significanat differences between time 0 and 365 at 22°C (p = 0.0244).

^†^ Significant differences between time 0 and 365 at 4°C (p = 0.0424).

**Fig 4:**
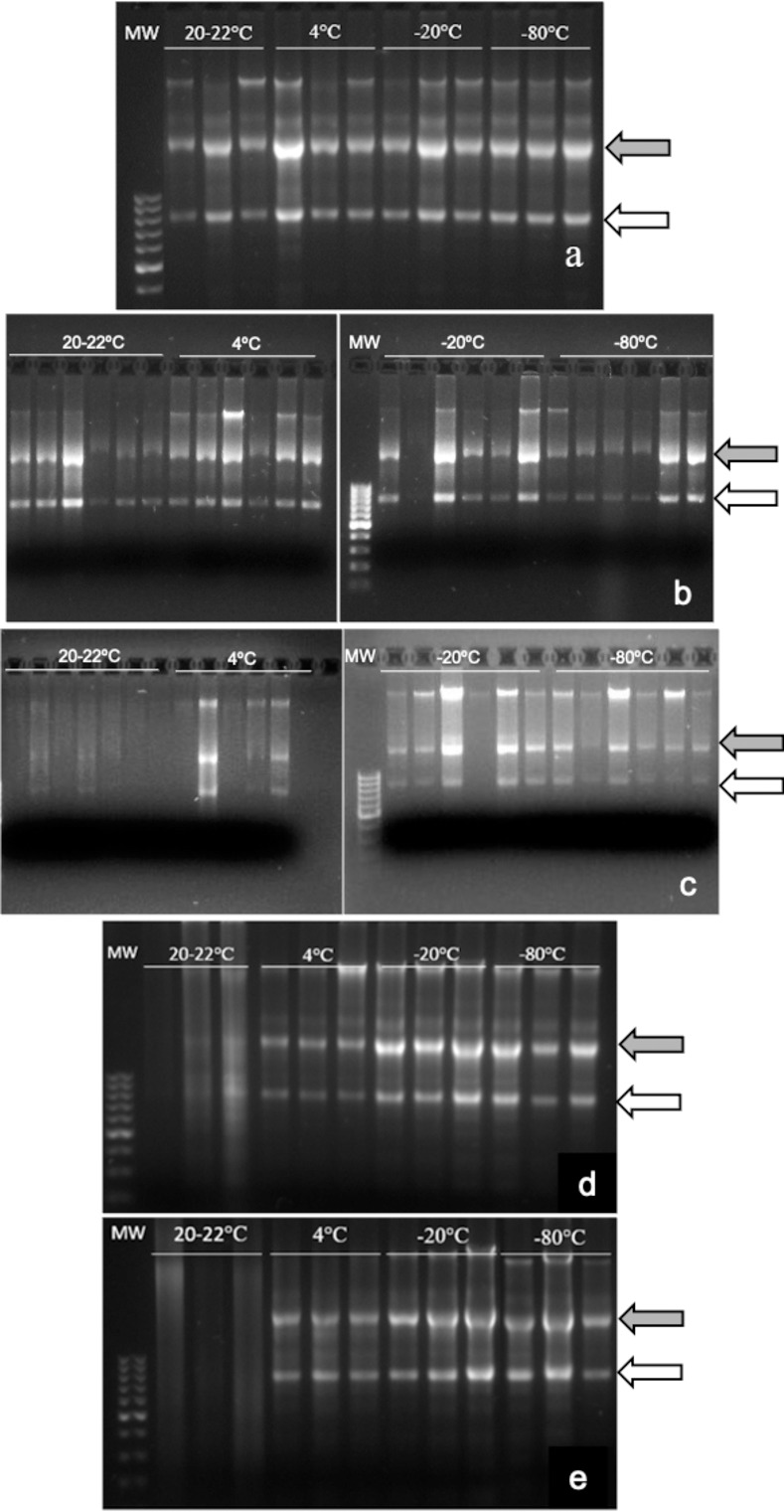
Agarose gel electrophoresis of testes samples stored in RNAlater^®^ at different temperatures for different times. Samples of testes were preserved in RNAlater^®^ at different temperatures for up to 365 days. **a.** Samples stored for 24 h (time 0), N = 9. **b.** Samples stored for 7 days, N = 6. **c**. Samples stored for 30 days, N = 6. **d**. Samples stored for 90 days, N = 3. **e**. Samples stored for 365 days, N = 3. MW: molecular weight marker. Grey arrow: rRNA28S; white arrow: rRNA18S.

After separation in agarose gels, samples stored for 24 h (time 0) and 7 days at different temperatures (Fig 4A and 4B) showed two clear bands representing the 28S and 18S subunits, indicating that RNA was not degraded. After 30 days, bands of subunits 28S and 18S of room temperature and 4°C samples were less evident (Fig 4C). For samples kept during 90 or 365 days at room temperature (Fig 4D and 4E), bands were not visible and smeared, meaning a loss of their RNA integrity. Two rRNA bands were observed in the rest of samples, showing that RNA integrity was well preserved.

#### 2.2. Protamine expression

In RNAlater^®^ samples, *Prm1* expression was similar after 24 h (time 0) and 7 days in all temperatures studied. After 30 days, samples stored at room temperature showed higher values of delta Ct *Prm1* than -80°C ones (3.92±0.36 *vs*. 1.92±0.39, respectively, p = 0.0425). Interestingly, these differences disappeared after 90 days of storage, but were marked after 365 days, being higher in room temperature (12.98±1.09) than -20°C (5.16±0.3, p = 0.0371) and -80°C (4.73±0.56, p = 0.0207) and 4°C samples (8.01±0.29) than -20°C (p = 0.0081) and -80°C ones (p = 0.0407) (see Table 2 in S2 File for data).

*Prm2* expression was similar in time-0 and day-7 samples stored at room temperature (5.46±0.91 and 4.53±0.38), 4°C (5.99±0.98 and 3.97±0.36), -20°C (5.27±1.2 and 3.39±0.57) and -80°C (5.13±1.32 and 2.48±0.85). After 30 days of storage, room temperature samples showed higher values (3.07±0.4) than -80°C ones (1.13±0.21, p = 0.0185), similar to was seen in *Prm1* expression. After 365 days, testes stored at room temperature showed higher values (12.65±0.85) than those stored at -20°C (5.56±0.55, p = 0.0121) and -80°C (3.99±0.48, p = 0.0076) (see Table 2 in S2 File for data).

**Fig 5:**
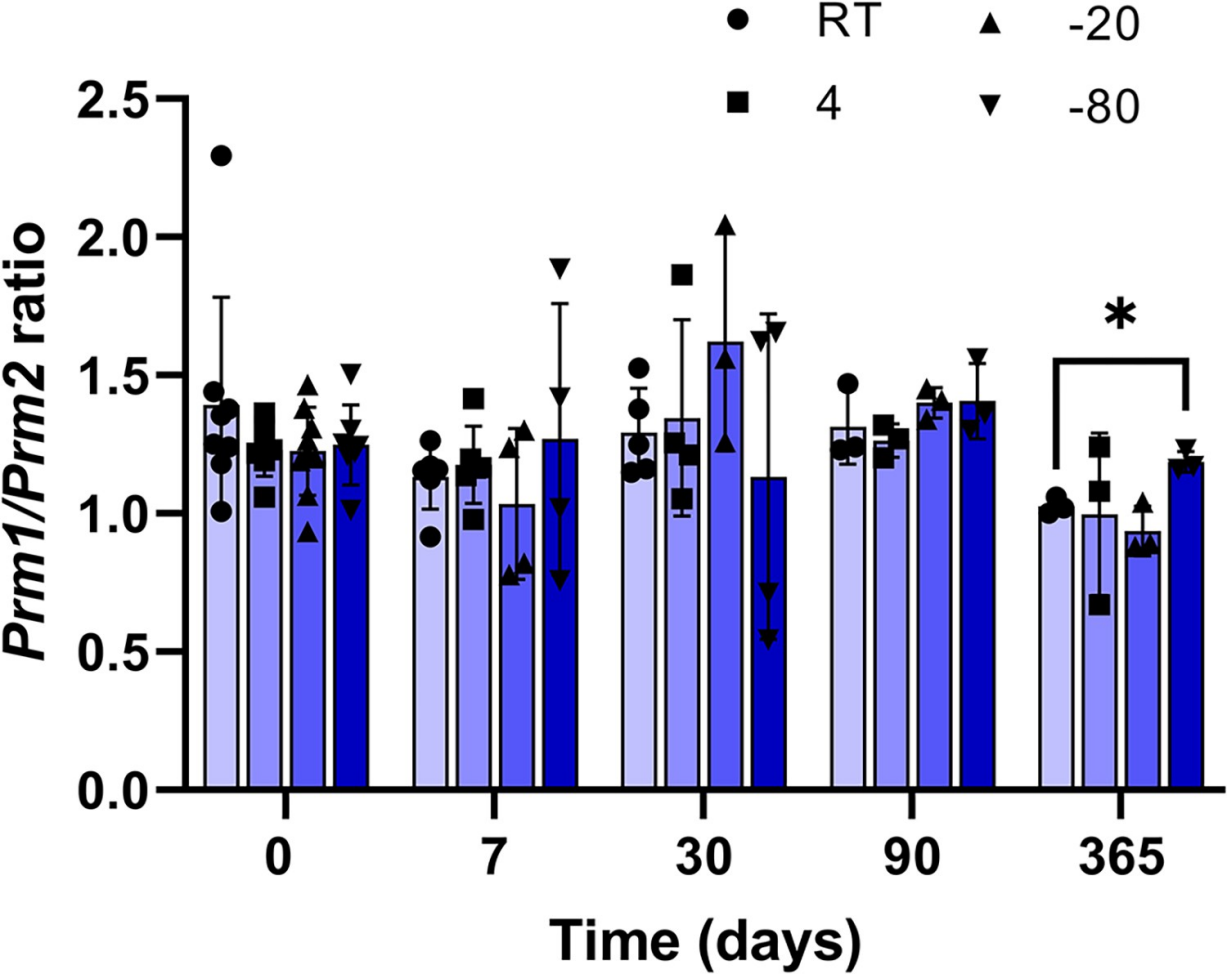
Protamine ratio (*Prm1/Prm2*) of testes samples stored in RNAlater^®^ at different temperatures for different times. Testes samples were preserved in RNAlater^®^ at different temperatures for up to 365 days. Light blue bars: room temperature (RT, 22°C); blue bars: 4°C; dark blue bars: -20°C; very dark blue bars: -80°C. *: significant differences (p = 0.0179) between different temperatures in the same time-point. Data are means±SEM; individual values are presented (N = 3–9).

The *Prm1/Prm2* ratio (Fig 5, see Table 3 in S2 File for data) was similar in samples stored at different temperatures in the different times tested, with the exception of the storage for 365 days, which showed differences between room temperature (1.03±0.02) and -80°C (1.19±0.02, p = 0.0179).

#### 2.3. Evaluation of the protocol for sample preservation with RNAlater^®^ at -80°C

Since gene expression decreased in samples stored at -80°C after 30 days, two methods of preservation of samples were compared, *i*.*e*., the commercial protocol (immersing the samples in RNAlater^®^ overnight (O/N) at 4°C, then removing the RNAlater^®^ and storing the samples at the desired temperature), and freezing samples immersed in RNAlater^®^ with no O/N incubation.

**Fig 6:**
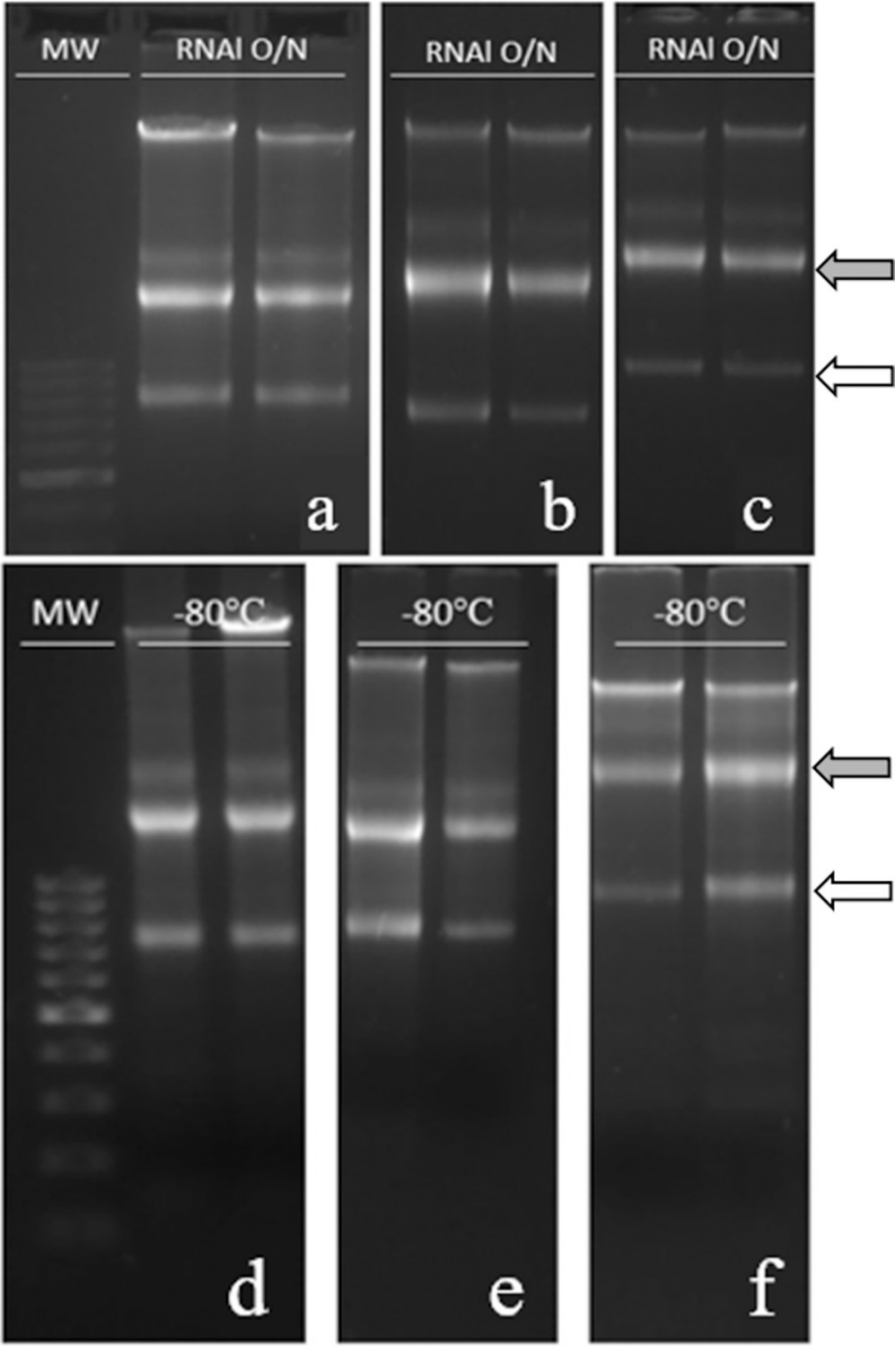
Agarose gel electrophoresis of testes samples stored at -80°C without or with removal of RNAlater^®^. Samples of testes were kept in RNAlater^®^ O/N or frozen directly at -80°C for different times. **a, b, c**: Samples kept O/N with RNAlater^®^ and then stored for 24 h (time 0), 7 or 30 days; **d, e, f**: Samples in RNAlater^®^ directly frozen to -80°C and stored for 24 h, 7 or 30 days. MW: molecular weight marker. Grey arrow: rRNA28S; white arrow: rRNA18S.

All samples showed high RNA purity and integrity, since the A260/A280 ratio value was close to 2 and RQI values were close to 10 (see Table 4 in S2 File for data). There were no significant differences between the different storage times, as well as between the different conditions of conservation. After separation in agarose gels, samples stored for 24 h (time 0) (Fig 6) showed two clear bands representing the 28S and 18S subunits in both treatments, indicating that RNA was not degraded in any of the samples. The same occurred for samples stored for 7 or 30 days (see Table 4 in S2 File for data).

The expression of *Prm1* and *Prm2* genes (see Table 5 in S2 File for data) was similar in all samples stored for 24 h or 7 days. In samples stored for 7 days, *Prm1* expression in samples incubated at 4°C O/N was higher but no significant (9.15±0.36) than frozen samples immersed in buffer (7.85±0.64). In contrast, in samples stored for 30 days, the expression was slightly higher but no significant in samples frozen immersed in buffer (8.44±0.01) than those incubated in buffer at 4°C O/N and then the buffer discarded (7.36±0.19). The same occurred for *Prm2* expression: there were no significant differences between the two storage methods nor between conditions.

### 3. Preservation of testes using snap-freezing and subsequent storage at -80°C or in liquid nitrogen

**Fig 7:**
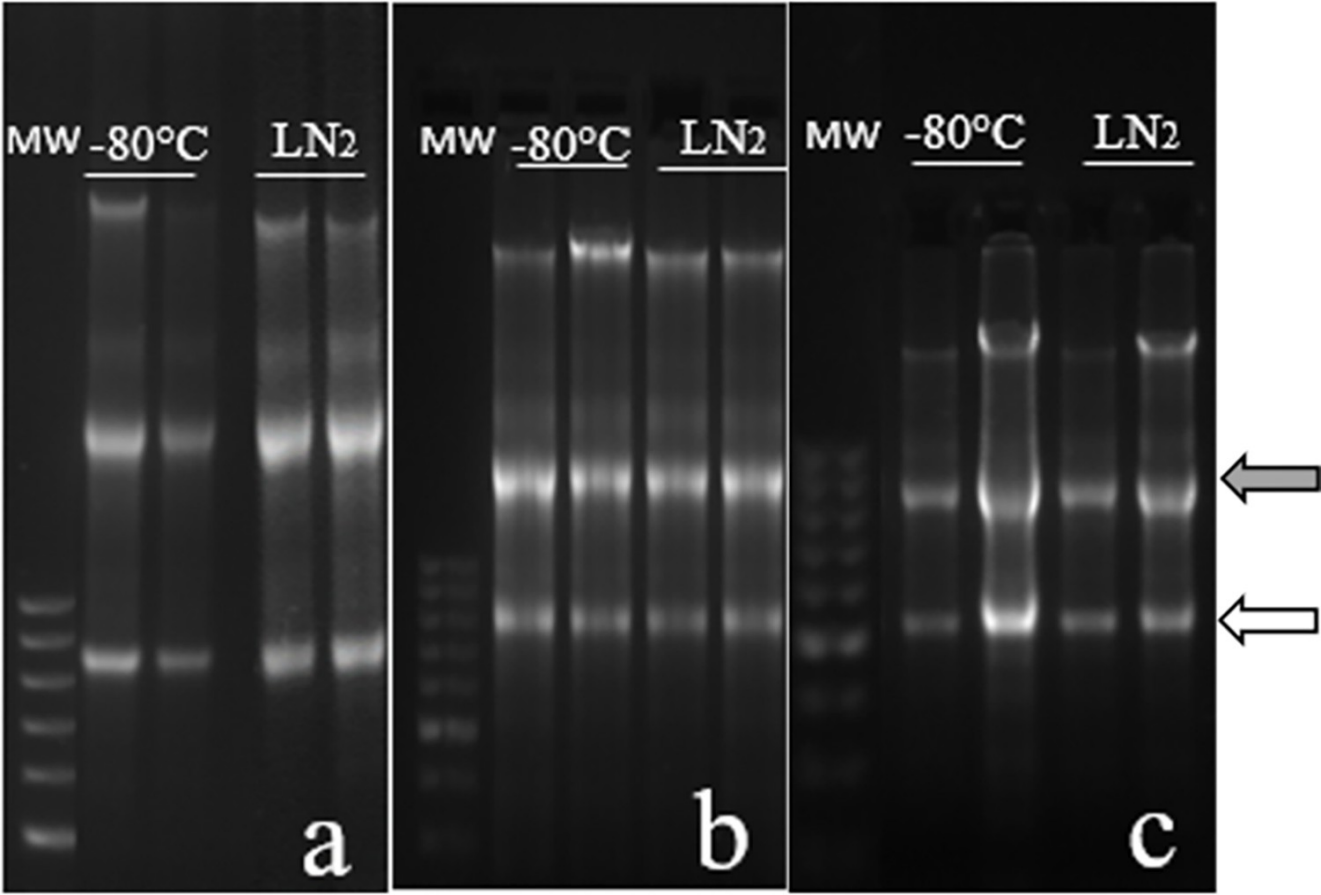
Agarose gel electrophoresis of testes samples snap-frozen and stored at different temperatures over time. Testes samples were snap-frozen and then preserved for different times at -80°C or in LN_2_. **a**: samples stored for 24 h (time 0); **b**: samples stored for 90 days; **c**: samples stored for 365 days. MW: molecular weight marker. Grey arrow: rRNA28S; white arrow: rRNA18S.

A high concentration of RNA was obtained from samples stored for 24 h (time 0), 90 days and 365 days in both conditions, and the RQI and 28S/18S ratio values were the highest (RQI = 10, see Table 1 in S3 File for data) in both situations. A260/280 ratio values were close to 2 (see Table 1 in S3 File for data), indicating that RNA purity was optimal in all samples at the different times studied. In 1.8% agarose gels (Fig 7), two clear bands representing the 28S and 18S rRNA subunits were observed for every time, regardless the temperature of storage (-80°C or LN_2_), suggesting that the RNA had been preserved intact.

When analyzing protamine expression (Table 2, see Table 2 in S2 File for data), no significant differences were observed between both storage methods for *Prm1* or *Prm2* expression. However, *Prm2* expression showed a significant decrease (p = 0.049) in samples stored in liquid nitrogen between time 0 (8.05±0.09) and 90 days (7.47±0.10). A non-statistically decrease was observed in the expression of both protamines after storage of the samples for 365 days. The difference observed in the *Prm1/Prm2* ratio was not significant.

**Table 2:** Protamine expression testes samples snap-frozen and stored at different temperatures over time.

Time (days)	Temperature	*Prm1* expression	*Prm2* expression	*Prm1/Prm2* ratio
0	Snap-freezing / -80°C	10.63±0.72	8.97±0.88	1.19±0.04
0	Snap-freezing / LN_2_	9.65±0.15	8.05±0.09*	1.2±0.03
90	Snap-freezing / -80°C	8.51±0.53	6.66±0.71	1.29±0.06
90	Snap-freezing / LN_2_	9.26±0.23	7.47±0.10*	1.24±0.01
365	Snap-freezing / -80°C	5.20±1.24	4.55±0.78	1.13±0.08
365	Snap-freezing / LN_2_	5.55±0.49	4.27±0.70	1.31±0.1

Testes samples were snap-frozen and then stored for 24 h (time 0), 90 or 365 days at -80°C or in liquid nitrogen (LN_2_).

*: significant differences between samples stored at the same temperature for different time points (p = 0.049). Data are means±SEM.

## Discussion

For the analysis of protamine gene expression, it is necessary that the tissue from which the sample is taken maintains an adequate RNA quality, that may be achieved by collecting the samples in different RNA-stabilizing buffers. The most commonly used buffer is RNAlater^®^, although this involves a significant cost when numerous samples are collected. A possible alternative is found in non-commercial buffers [9] which appear to have an equivalent ability to preserve RNA and DNA of somatic tissues. This study analyzed protamine expression and RNA quality in testes samples stored at different conditions of buffer and temperature for up to 1 year. Testes samples were processed without completely removing the tunica albuginea since it has been reported that cell viability and the structure integrity is better maintained if the tunica is partially preserved [16].

We first analyzed if the NAP buffer was reliable for the preservation of testicular tissue, analyzing RNA quality and purity, in comparison with liver tissue [9]. NAP buffer at -80°C conserved high RNA integrity and purity in both testes and liver, suggesting that this buffer may be used instead of RNAlater^®^ in testes. Nunes *et al*. [20] obtained negative results using NAP buffer as a preservative, as the DNA recovered from the samples did not have sufficient concentration and purity for further molecular analyses. This disagreement may be due to the sample preserved–whereas the present study used animal tissue, the mentioned study used isolated cells, which may have limited genetic material preservation.

After corroborating the good performance of NAP buffer with testis tissue, and in order to facilitate the collection of samples under field conditions, we studied both RNA integrity and *Prm* expression in testes kept in NAP buffer and RNAlater^®^ at room temperature for 7 days. When working in the field, it could be difficult to achieve temperatures different from room temperature, and normally the maximum time from collection to get to the laboratory is approximately a week. As mentioned above, the most commonly used RNA stabilizing buffer is the commercial RNAlater^®^, capable of preserving RNA at room temperature for 7 days, although some studies [21] reported that RNAlater^®^ can substantially alter the physiology of samples and impact gene expression non-randomly. Our results showed that RNA purity and integrity was maintained when samples were preserved in RNAlater^®^ or in NAP buffer for 7 days. The results obtained for *Prm1/Prm2* ratio were close to 1 in samples analyzed at times 0 and 7 days stored at room temperature, in agreement with previous studies [22] where the ratio of *Prm1/Prm2* in *Mus musculus* was 0.95. These results suggest that it is possible to obtain reliable results of protamine expression when collecting and storing testes from wild animals for 7 days at room temperature in both RNAlater^®^ and NAP buffer.

To assess the effect of different temperatures on RNA quality and *Prm* expression, we stored testes samples in RNAlater^®^ for 365 days at different temperatures. In agreement with the results obtained by Camacho-Sanchez *et al*. [9] in somatic tissues, the testicular samples stored at -80°C showed high RNA purity and integrity. Interestingly, RQI values and the ratio 28S/18S from samples stored for one year at 4°C and -20°C also showed good results, suggesting the possibility of storing testes at these temperatures when an ultra-freezer is not available, in accordance with the conclusions of Van Cise *et al*. [23] who indicate that for long-term preservation, in the absence of an ultra-freezer, the best option is to keep the tissue in a liquid preservative and freeze the samples at -20°C. Moreover, *Prm1/Prm2* ratios in samples stored at the temperatures tested up to 90 days were consistent and similar between them. After 365 days of storage, this ratio decreased in room temperature samples, probably corresponding with the lowest RNA integrity found in these tissues, as reported previously [10]. These results are in accordance with those which compared the integrity of somatic tissue RNA immersed in RNAlater^®^ when stored at 4°C *versus* room temperature, obtaining favorable results for the lower temperature [24]. In contrast, recent studies [25] reported a high integrity and quality of genomic DNA after storage for extended periods of time at room temperature in a homemade buffer, maybe because of different composition with the buffers used in the present study. In general, the expression levels were constant and relate with previous results from our laboratory [5], which revealed that the expression of these genes did not differ between individuals of the same species.

The RNAlater^®^ commercial protocol indicates that samples to be stored at room temperature or 4°C should remain immersed in the stabilizing buffer. Conversely, for those to be stored at -20°C or -80°C, the RNAlater^®^ should be removed after O/N incubation with the buffer. Since results showed that protamine expression was lower after one year in samples stored at -80°C than in those stored at 4°C, an additional experiment was performed. Protamine expression was analyzed in samples stored at -80°C immersed in RNAlater^®^, or stored at -80°C after removal of RNAlater^®^. Samples were analyzed after 24 h, 7 and 30 days of storage. Although no significant differences were obtained between the two methods of sample preservation, expression was slightly higher and more stable for samples stored at -80°C immersed in RNAlater^®^. These results could explain why gene expression results in samples stored at 4°C were higher than in those stored at -80°C, since those at 4°C and room temperature remained immersed in RNAlater^®^ throughout the experiment, although further studies are necessary to corroborate these results. Our results are in accordance with other studies in tumor tissue [12], liver [13] or other somatic tissues [14], which found that freezing samples at -80°C without RNAlater^®^ leads to a decrease in RNA quality, whereas the immersion in RNAlater^®^ prevents damage to the tissue that can affect RNA quality.

Finally, we also tested the storage at -80°C or in LN_2_, after snap-freezing the testes. In agreement with results by Perlmutter *et al*. [10], who found that this technique is the best way of preserving RNA with subsequent storage at -80°C or in LN_2_, we obtained high RNA quality and protamine expression with both storage methods. This is a good method for long-term preservation and storage, but sometimes it is a difficult technique to use when samples are collected under field conditions [26, 27]. Dumond et al. [28] evaluated different protocols for freezing testicular tissue to allow the germ cell preservation and its subsequent completion of spermatogenesis by in vitro maturation. They concluded that the best protocol was a modified solid surface vitrification (see [28]). Although our aim was not maintaining the cell viability or the tissue structure but the analysis of gene expression, these important results of testes vitrification cannot be ignored.

In conclusion, for the preservation of testis samples, NAP buffer acts as a stabilizing buffer with the same efficiency as the commercial RNAlater^®^. Regarding the different temperatures of storage in RNAlater^®^, 4°C and -20°C preserved the integrity and purity of RNA and protein gene expression to a degree similar to those stored at -80°C, even after one year of storage. These results open new approaches for the collection and storage of testes when ultra-freezers are not available or under field conditions. Furthermore, although samples immersed in RNAlater^®^ and stored at room temperature for 90 days showed lower RNA quality and integrity than those stored in the freezer and ultra-freezer, the ratio between protamines was not affected. Skrypina *et al*. [29] studied the possibility of obtaining cDNA for subsequent gene expression analysis from samples with RNA degradation, analyzed from the 28S/18S ratio in agarose gel. They concluded that gene expression by RT-PCR may not be affected by unspecific degradation of total RNA, when using random primer for cDNA synthesis. In our study, even oligo(dT) primers were used, protamine expression appeared to remain the same regardless of the temperature of storage up to 90 days. Finally, the temperature of storage after snap-freezing (-80°C or LN_2_) maintained RNA quality and protamine expression, facilitating the choice of storage method depending on the facilities available. These results open new possibilities for sample collection in the field for subsequent studies of gene expression, using different buffers and storage temperatures.

## Supporting information

S1 Fig Agarose gel electrophoresis of liver and testis samples stored 24 hours at different temperatures. 1 and 2: Liver samples preserved in RNAlater^®^, stored at -80°C; 5 and 6: liver samples preserved in NAP buffer, stored at -80°C; 9 and 10: testis samples preserved in RNAlater^®^, stored at room temperature (RT); 11 and 12: testis samples preserved in NAP buffer, stored at RT; 13 and 14: testis samples snap-frozen and then stored at -80°C; MW: molecular weight marker.
(TIF)

S2 Fig Agarose gel electrophoresis of testis samples stored 24 hours at -80°C. 1 and 2: Testis samples preserved in RNAlater^®^; 3 and 4: testis samples preserved in NAP buffer; MW: molecular weight marker.
(TIF)

S3 Fig Agarose gel electrophoresis of testis samples stored 7 days at room temperature (RT) (20–22°C). 1 and 2: testis samples preserved in RNAlater^®^; 3 and 4: testis samples preserved in NAP buffer; MW: molecular weight marker.
(TIF)

S4 Fig Agarose gel electrophoresis of testis samples preserved in RNAlater^®^ for 24 hours at different temperatures. 1, 2 and 3: testis samples stored at RT; 4,5 and 6: testis samples stored at 4°C; 7, 8 and 9: testis samples stored at -20°C; 10, 11 and 12: testis samples stored at -80°C; MW: molecular weight marker.
(TIF)

S5 Fig Agarose gel electrophoresis of testis samples preserved in RNAlater^®^ for 7 days at RT or 4°C. 1–6: testis samples stored at RT; 7–12: testis samples stored at 4°C; MW: molecular weight marker.
(TIF)

S6 Fig Agarose gel electrophoresis of testis samples preserved in RNAlater^®^ for 7 days at -20°C or -80°C. 1–6: testis samples stored at -20°C; 7–12: testis samples stored at -80°C; MW: molecular weight marker.
(TIF)

S7 Fig Agarose gel electrophoresis of testis samples preserved in RNAlater^®^ for 30 days at RT or 4°C. 1–6: testis samples stored at RT; 7–12: testis samples stored at 4°C; MW: molecular weight marker.
(TIF)

S8 Fig Agarose gel electrophoresis of testis samples preserved in RNAlater^®^ for 30 days at -20°C or -80°C. 1–6: testis samples stored at -20°C; 7–12: testis samples stored at -80°C; MW: molecular weight marker.
(TIF)

S9 Fig Agarose gel electrophoresis of testis samples preserved in RNAlater^®^ for 90 days at different temperatures. 1, 2 and 3: testis samples stored at RT; 4,5 and 6: testis samples stored at 4°C; 7, 8 and 9: testis samples stored at -20°C; 10, 11 and 12: testis samples stored at -80°C; MW: molecular weight marker.
(TIF)

S10 Fig Agarose gel electrophoresis of testis samples preserved in RNAlater^®^ for 365 days at different temperatures. 1, 2 and 3: testis samples stored at RT; 4,5 and 6: testis samples stored at 4°C; 7, 8 and 9: testis samples stored at -20°C; MW: molecular weight marker.
(TIF)

S11 Fig Agarose gel electrophoresis of testis samples stored for 365 days at different storage conditions. 1 and 2: testis samples snap-frozen and then stored at -80°C; 3 and 4: testis samples snap-frozen and then stored in liquid nitrogen (LN2); 5, 6 and 7: testis samples preserved in RNAlater^®^ and stored at -80°C; MW: molecular weight marker.
(TIF)

S1 File **Table 1.** Raw data table from the RNA quality analysis of testes and liver samples, preserved in RNAlater^®^ or NAP buffer at -80°C for 24 hours. **Table 2.** Raw data table from the RNA quality analysis of testes samples, preserved in RNAlater^®^ or NAP buffer at RT, 24 hours (time 0) or 7 days. **Table 3.** Raw data table from protamine expression analysis of testes samples, preserved in RNAlater^®^ or NAP buffer at RT, 24 hours (time 0) or 7 days. **Table 4.** Raw data table from protamine ratio, analysis of testes samples, preserved in RNAlater^®^ or NAP buffer at RT, 24 hours (time 0) or 7 days.
(XLSX)

S2 File **Table 1.** Raw data table from the RNA quality analysis of testes samples, preserved in RNAlater^®^ at different temperatures for up to 365 days. **Table 2.** Raw data table from the protamine expression analysis of testes samples, preserved in RNAlater^®^ at different temperatures for up to 365 days. **Table 3**. Raw data table from the protamine ratio analysis of testes samples, preserved in RNAlater^®^ at different temperatures for up to 365 days. **Table 4.** Raw data table from the RNA quality analysis of testes samples, stored at -80°C without or with removal of RNAlater^®^. **Table 5.** Raw data table from the protamine expression analysis of testes samples, stored at -80°C without or with removal of RNAlater^®^.
(XLSX)

S3 File **Table 1.** Raw data table from the RNA quality analysis of testes samples snap-frozen and stored at -80°C or in LN2 for up to 365 days. **Table 2.** Raw data table from the protamine expression analysis of testes samples snap-frozen and stored at -80°C or in LN2 for up to 365 days.
(XLSX)

S1 Raw images (PDF)
