# Supplementary figures and images for: RNA quality and protamine gene expression after storage of mouse testes under different conditions
Source: PLoS One. 2024 Nov 21;19(11):e0314013. doi: 10.1371/journal.pone.0314013 (PMC11581210; doi:10.1371/journal.pone.0314013)

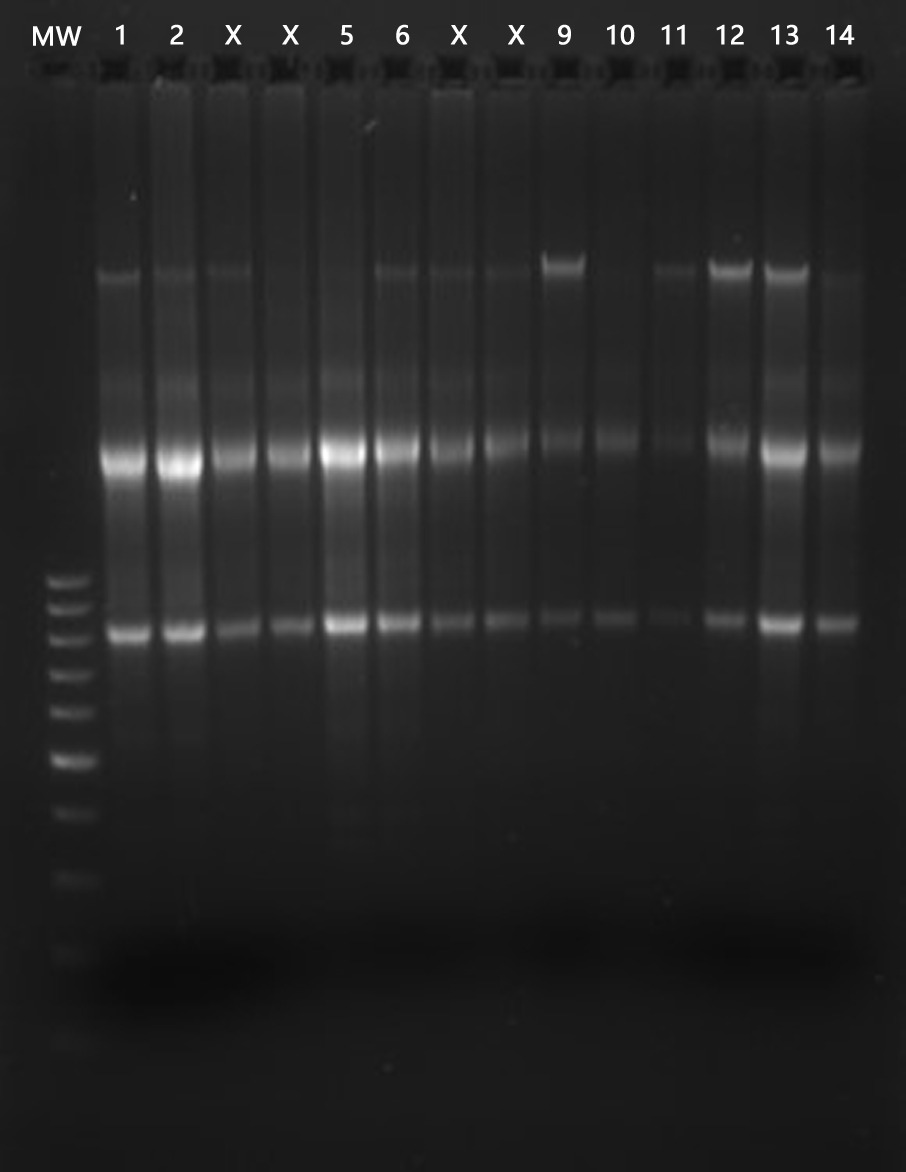

Supplement: S1 Fig — 1 and 2: Liver samples preserved in RNAlater®, stored at -80°C; 5 and 6: liver samples preserved in NAP buffer, stored at -80°C; 9 and 10: testis samples preserved in RNAlater®, stored at room temperature (RT); 11 and 12: testis samples preserved in NAP buffer, stored at RT; 13 and 14: testis samples snap-frozen and then stored at -80°C; MW: molecular weight marker. (TIF) [file pone.0314013.s001.tif]

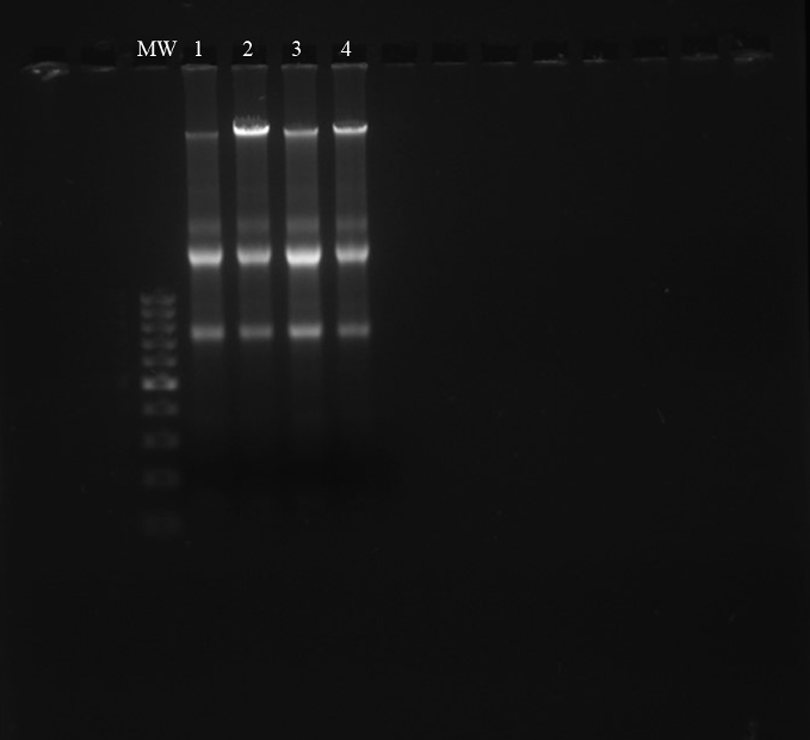

Supplement: S2 Fig — 1 and 2: Testis samples preserved in RNAlater®; 3 and 4: testis samples preserved in NAP buffer; MW: molecular weight marker. (TIF) [file pone.0314013.s002.tif]

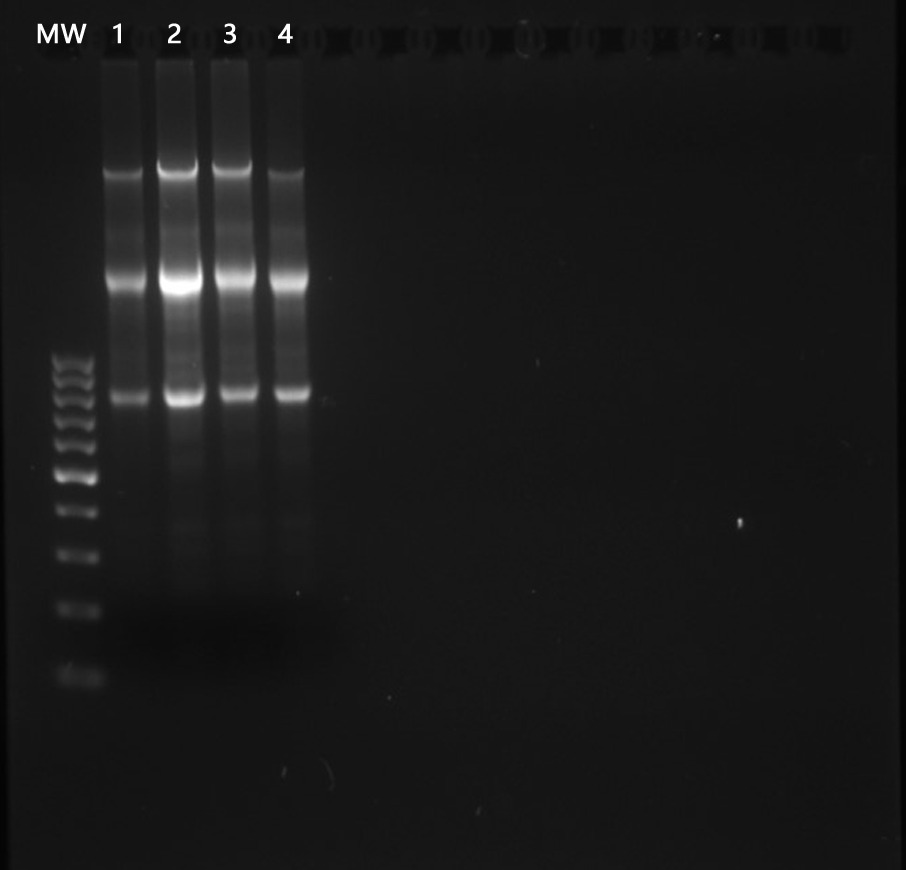

Supplement: S3 Fig — 1 and 2: testis samples preserved in RNAlater®; 3 and 4: testis samples preserved in NAP buffer; MW: molecular weight marker. (TIF) [file pone.0314013.s003.tif]

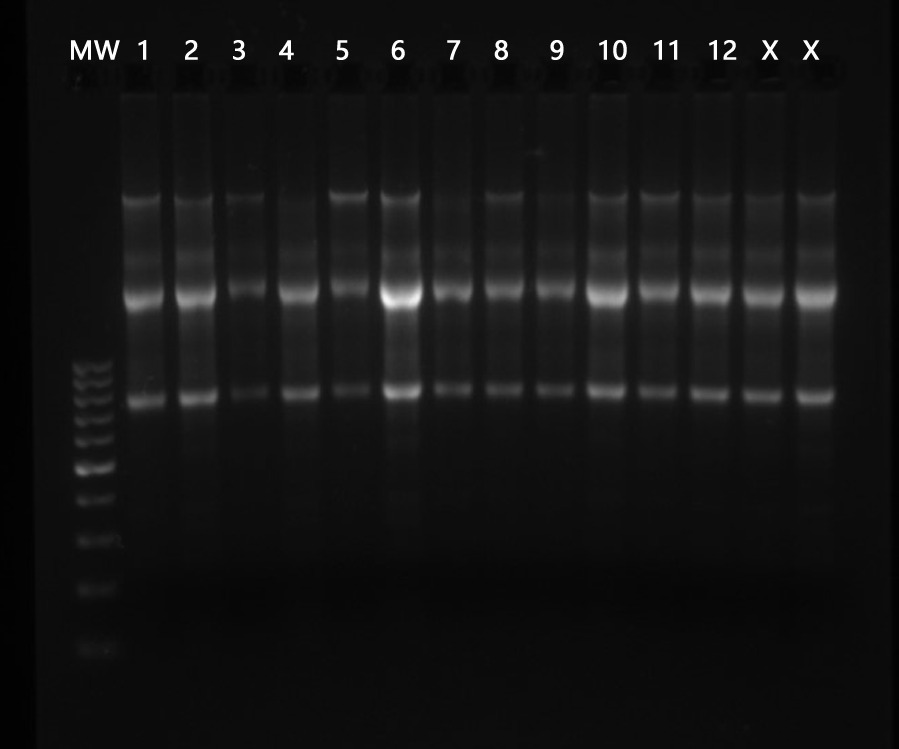

Supplement: S4 Fig — 1, 2 and 3: testis samples stored at RT; 4,5 and 6: testis samples stored at 4°C; 7, 8 and 9: testis samples stored at -20°C; 10, 11 and 12: testis samples stored at -80°C; MW: molecular weight marker. (TIF) [file pone.0314013.s004.tif]

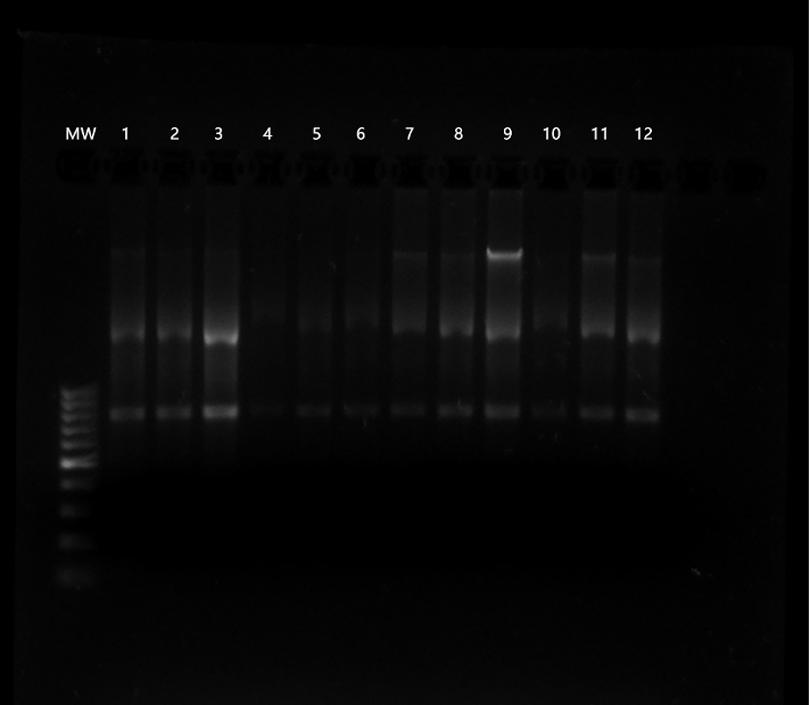

Supplement: S5 Fig — 1–6: testis samples stored at RT; 7–12: testis samples stored at 4°C; MW: molecular weight marker. (TIF) [file pone.0314013.s005.tif]

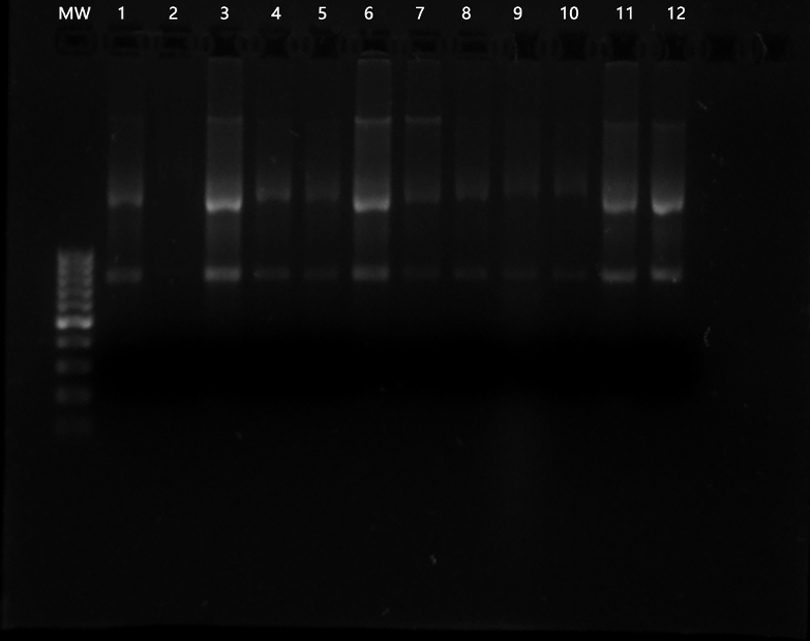

Supplement: S6 Fig — 1–6: testis samples stored at -20°C; 7–12: testis samples stored at -80°C; MW: molecular weight marker. (TIF) [file pone.0314013.s006.tif]

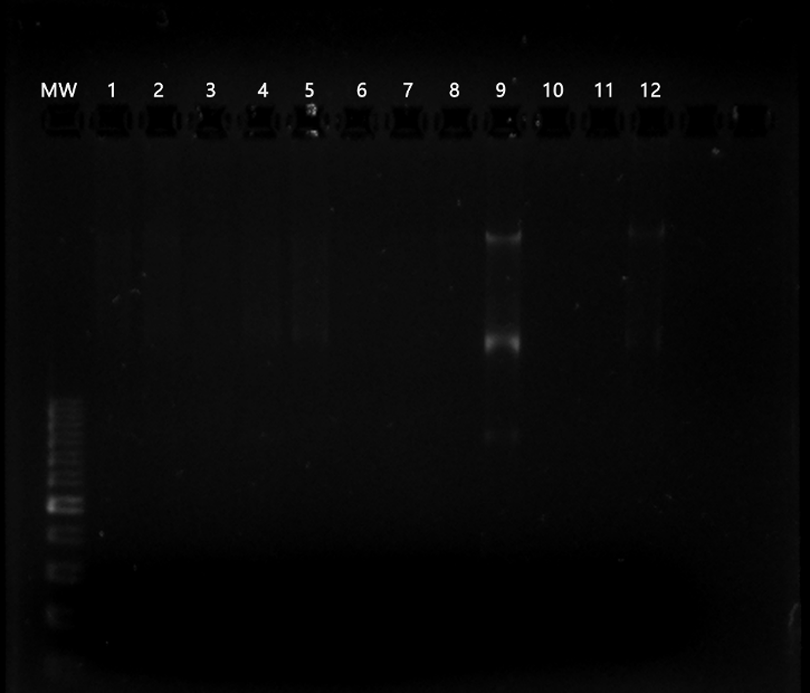

Supplement: S7 Fig — 1–6: testis samples stored at RT; 7–12: testis samples stored at 4°C; MW: molecular weight marker. (TIF) [file pone.0314013.s007.tif]

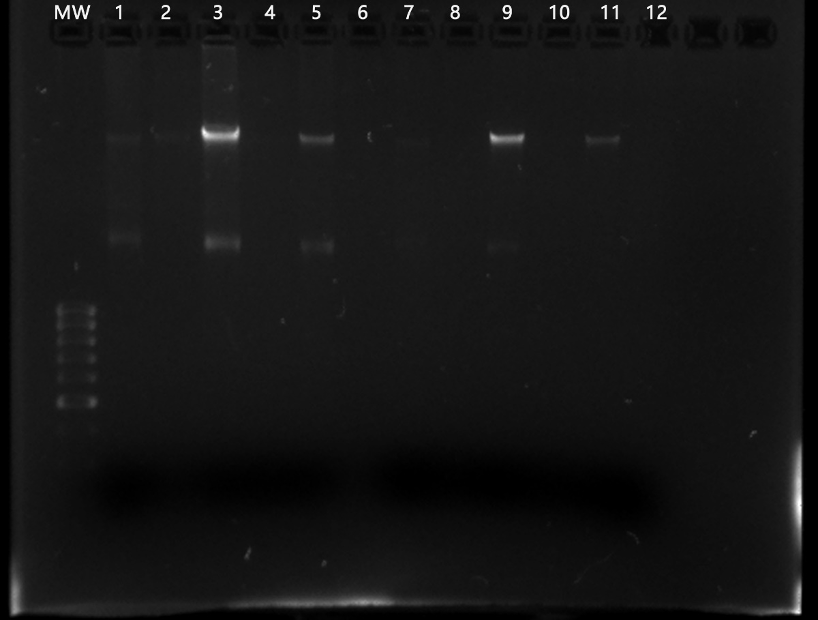

Supplement: S8 Fig — 1–6: testis samples stored at -20°C; 7–12: testis samples stored at -80°C; MW: molecular weight marker. (TIF) [file pone.0314013.s008.tif]

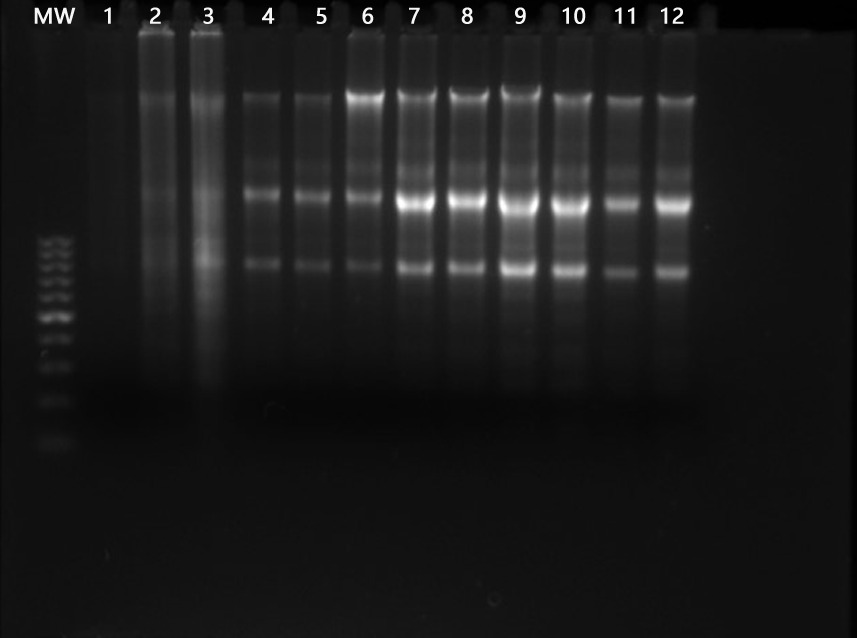

Supplement: S9 Fig — 1, 2 and 3: testis samples stored at RT; 4,5 and 6: testis samples stored at 4°C; 7, 8 and 9: testis samples stored at -20°C; 10, 11 and 12: testis samples stored at -80°C; MW: molecular weight marker. (TIF) [file pone.0314013.s009.tif]

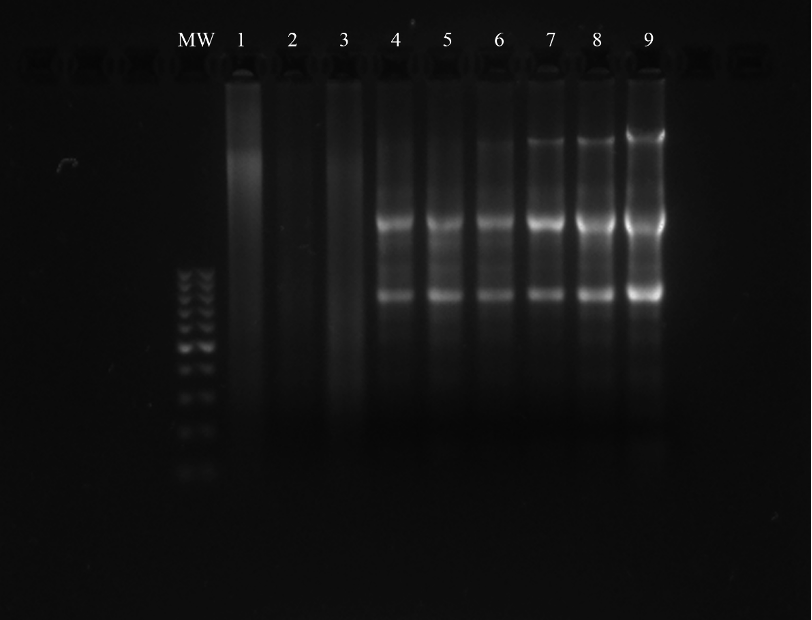

Supplement: S10 Fig — 1, 2 and 3: testis samples stored at RT; 4,5 and 6: testis samples stored at 4°C; 7, 8 and 9: testis samples stored at -20°C; MW: molecular weight marker. (TIF) [file pone.0314013.s010.tif]

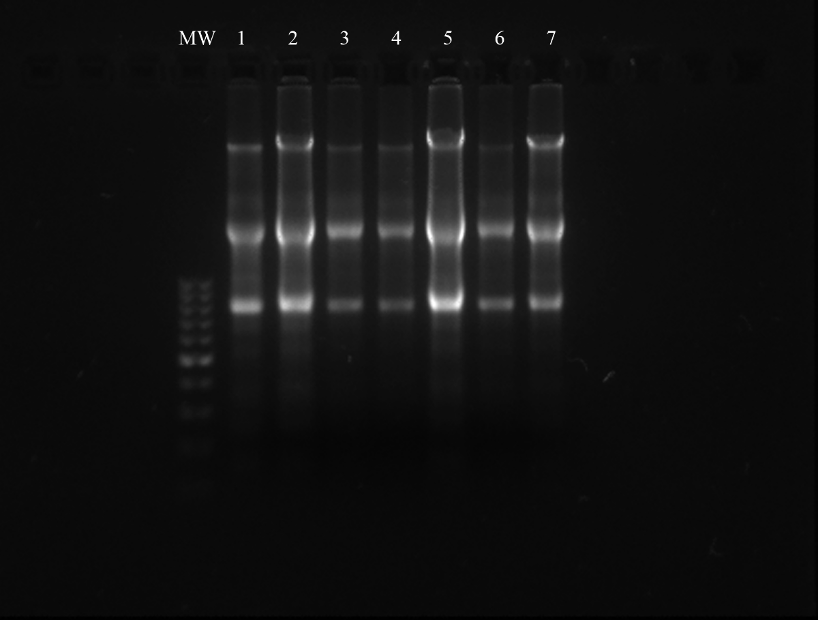

Supplement: S11 Fig — 1 and 2: testis samples snap-frozen and then stored at -80°C; 3 and 4: testis samples snap-frozen and then stored in liquid nitrogen (LN2); 5, 6 and 7: testis samples preserved in RNAlater® and stored at -80°C; MW: molecular weight marker. (TIF) [file pone.0314013.s011.tif]
