# Supplementary material for: RNA quality and protamine gene expression after storage of mouse testes under different conditions
Source: PLoS One. 2024 Nov 21;19(11):e0314013. doi: 10.1371/journal.pone.0314013 (PMC11581210; doi:10.1371/journal.pone.0314013)
Supplement: S1 Raw images — (PDF) [file pone.0314013.s015.pdf]

RNA quality and protamine gene expression after storage of mouse testes under different conditions

Nerea Latorre[1], Beatriz A. Dorda[2], Isabel Rey[2], Eduardo R. S. Roldan[1], Ana Sanchez-Rodriguez[1*]

[1]Department of Biodiversity and Evolutionary Biology, Museo Nacional de Ciencias Naturales (CSIC), Madrid, Spain.

[2]Tissues and DNA Collection, Museo Nacional de Ciencias Naturales (CSIC), Madrid, Spain.

* Corresponding author

E-mail: anasanchez@mncn.csic.es (ASR) ORCID: 0000-0002-3724-9470

Enumeration:

S1 Fig 1. Agarose gel electrophoresis of liver and testis samples stored 24 hours at different temperatures.

S2 Fig. Agarose gel electrophoresis of testis samples stored 24 hours at -80 ºC.

S3 Fig. Agarose gel electrophoresis of testis samples stored 7 days at room temperature (RT) (20-22 ºC).

S4 Fig. Agarose gel electrophoresis of testis samples preserved in RNAlater® for 24 hours at different temperatures.

S5 Fig. Agarose gel electrophoresis of testis samples preserved in RNAlater® for 7 days at RT or 4 ºC.

S6 Fig. Agarose gel electrophoresis of testis samples preserved in RNAlater® for 7 days at -20 ºC or -80 ºC.

S7 Fig. Agarose gel electrophoresis of testis samples preserved in RNAlater® for 30 days at RT or 4 ºC.

S8 Fig. Agarose gel electrophoresis of testis samples preserved in RNAlater® for 30 days at -20 ºC or -80 ºC.

S9 Fig. Agarose gel electrophoresis of testis samples preserved in RNAlater® for 90 days at different temperatures.

S10 Fig. Agarose gel electrophoresis of testis samples preserved in RNAlater® for 365 days at different temperatures.

S11 Fig. Agarose gel electrophoresis of testis samples stored for 365 days at different storage conditions.

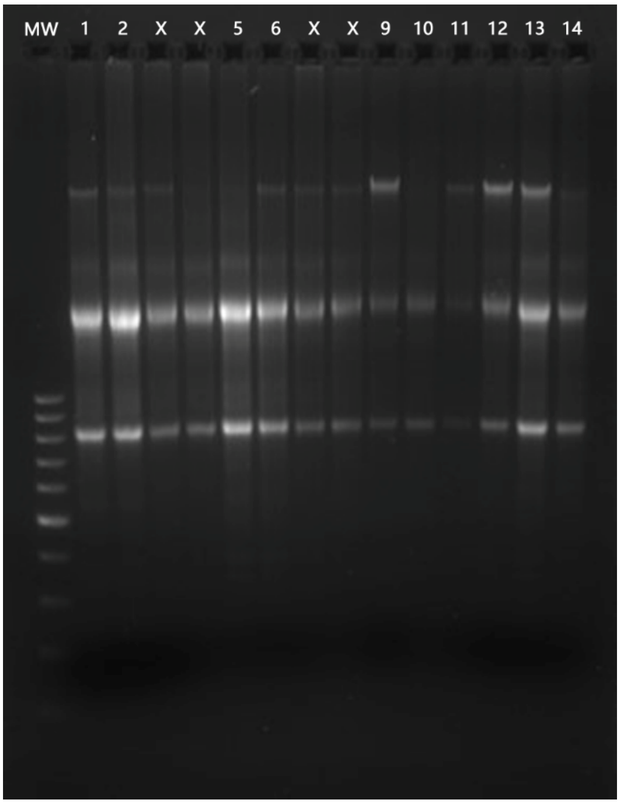

S1 Fig. Agarose gel electrophoresis of liver and testis samples stored 24 hours at different temperatures. 1 and 2: Liver samples preserved in RNAlater®, stored at -80 ºC; 5 and 6: liver samples preserved in NAP buffer, stored at -80 ºC; 9 and 10: testis samples preserved in RNAlater®, stored at room temperature (RT); 11 and 12: testis samples preserved in NAP buffer, stored at RT; 13 and 14: testis samples snap-frozen and then stored at -80 ºC; MW: molecular weight marker.

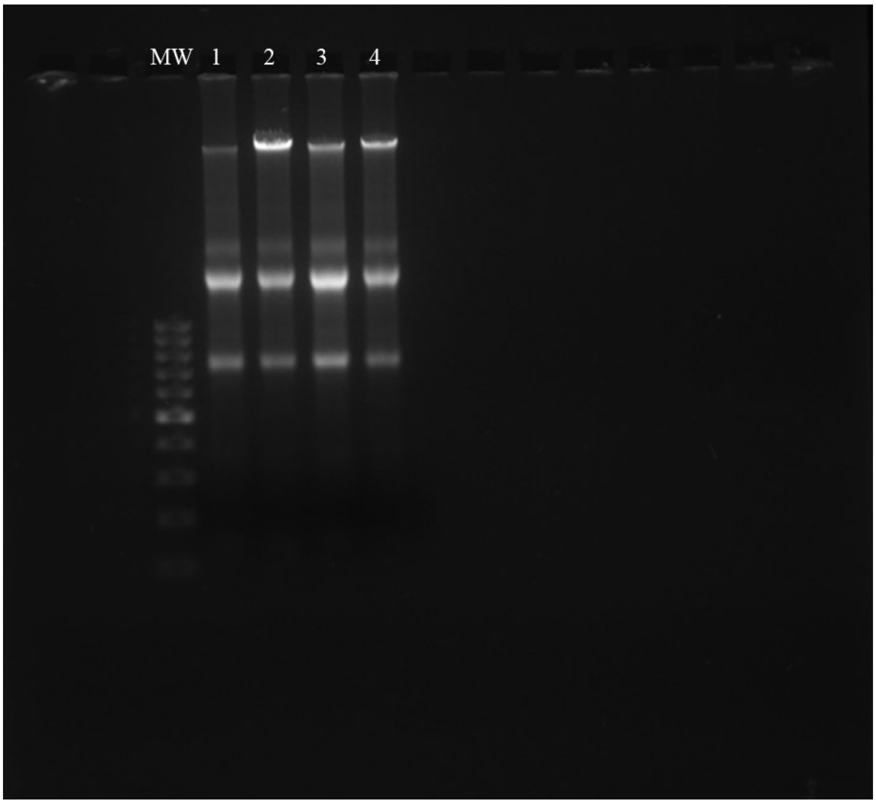

S2 Fig. Agarose gel electrophoresis of testis samples stored 24 hours at -80 ºC. 1 and 2: Testis samples preserved in RNAlater®; 3 and 4: testis samples preserved in NAP buffer; MW: molecular weight marker.

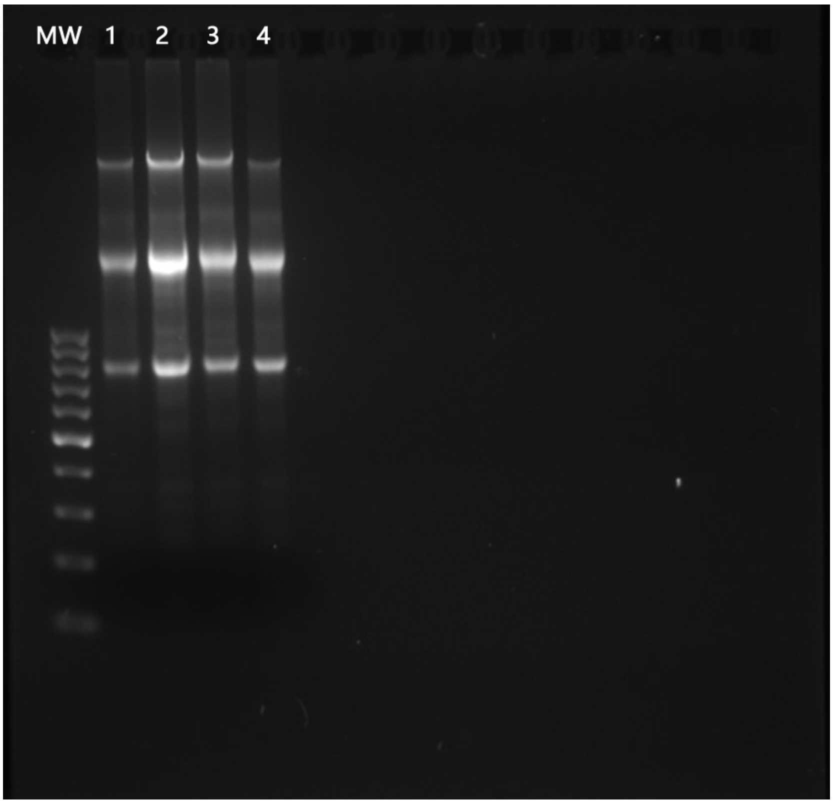

S3 Fig. Agarose gel electrophoresis of testis samples stored 7 days at room temperature (RT) (20-22 ºC). 1 and 2: testis samples preserved in RNAlater®; 3 and 4: testis samples preserved in NAP buffer; MW: molecular weight marker.

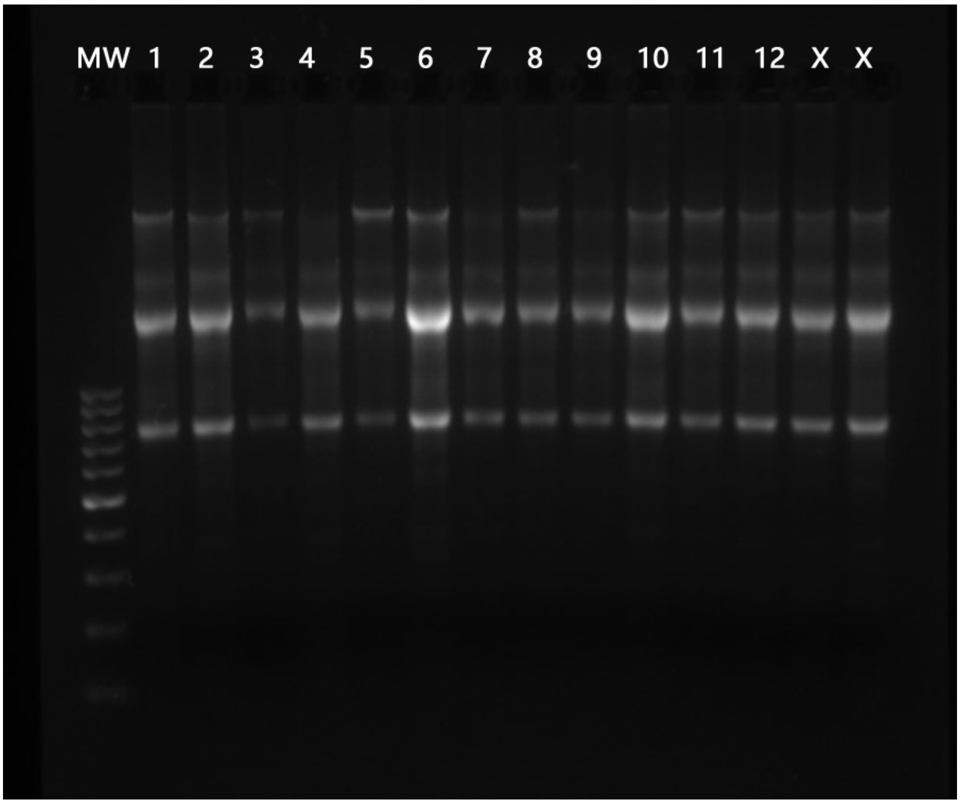

S4 Fig. Agarose gel electrophoresis of testis samples preserved in RNAlater® for 24 hours at different temperatures. 1, 2 and 3: testis samples stored at RT; 4,5 and 6: testis samples stored at 4 ºC; 7, 8 and 9: testis samples stored at -20 ºC; 10, 11 and 12: testis samples stored at -80 ºC; MW: molecular weight marker.

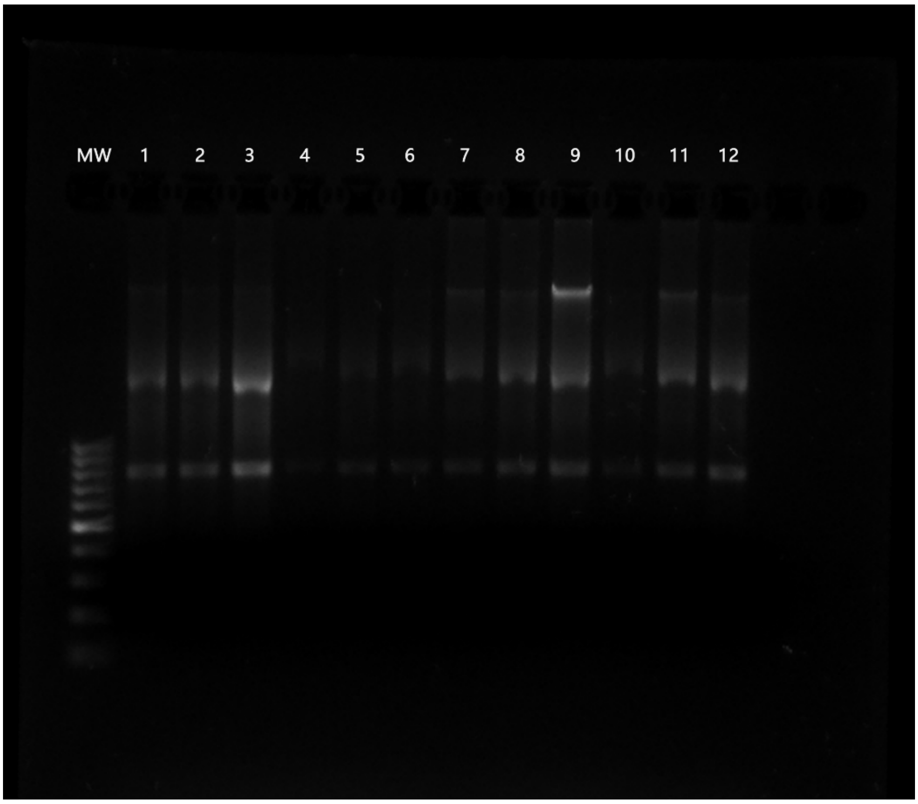

S5 Fig. Agarose gel electrophoresis of testis samples preserved in RNAlater® for 7 days at RT or 4 ºC. 1-6: testis samples stored at RT; 7-12: testis samples stored at 4 ºC; MW: molecular weight marker.

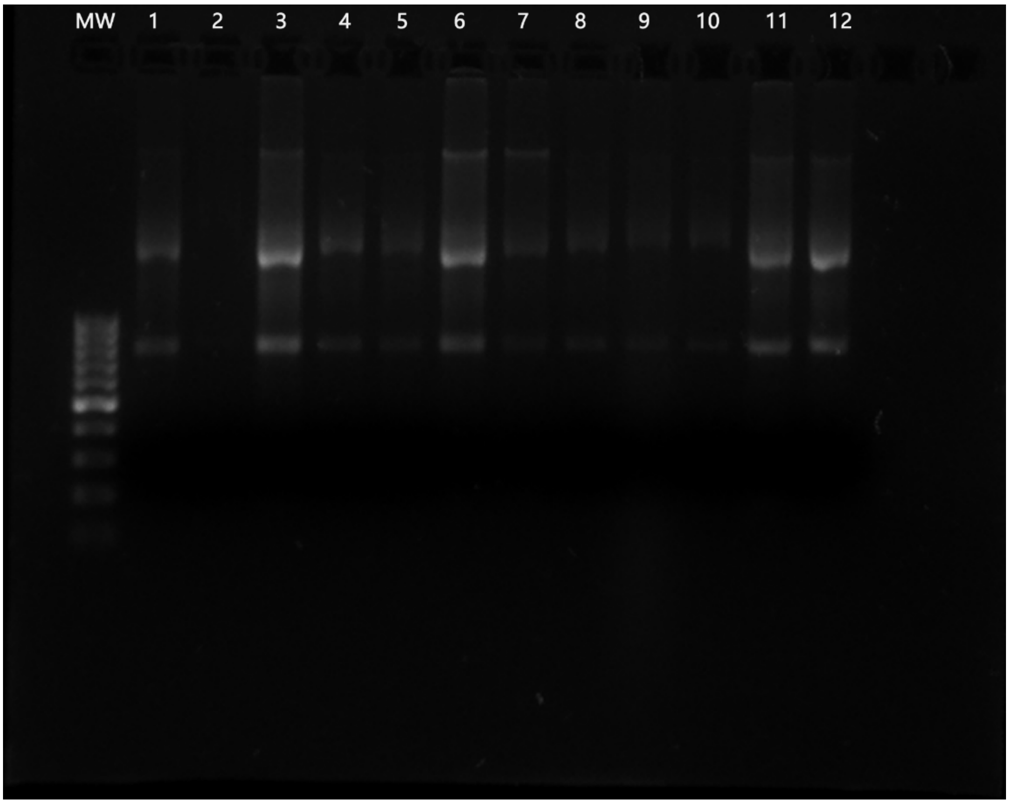

S6 Fig. Agarose gel electrophoresis of testis samples preserved in RNAlater® for 7 days at -20 ºC or -80 ºC. 1-6: testis samples stored at -20 ºC; 7-12: testis samples stored at -80 ºC; MW: molecular weight marker.

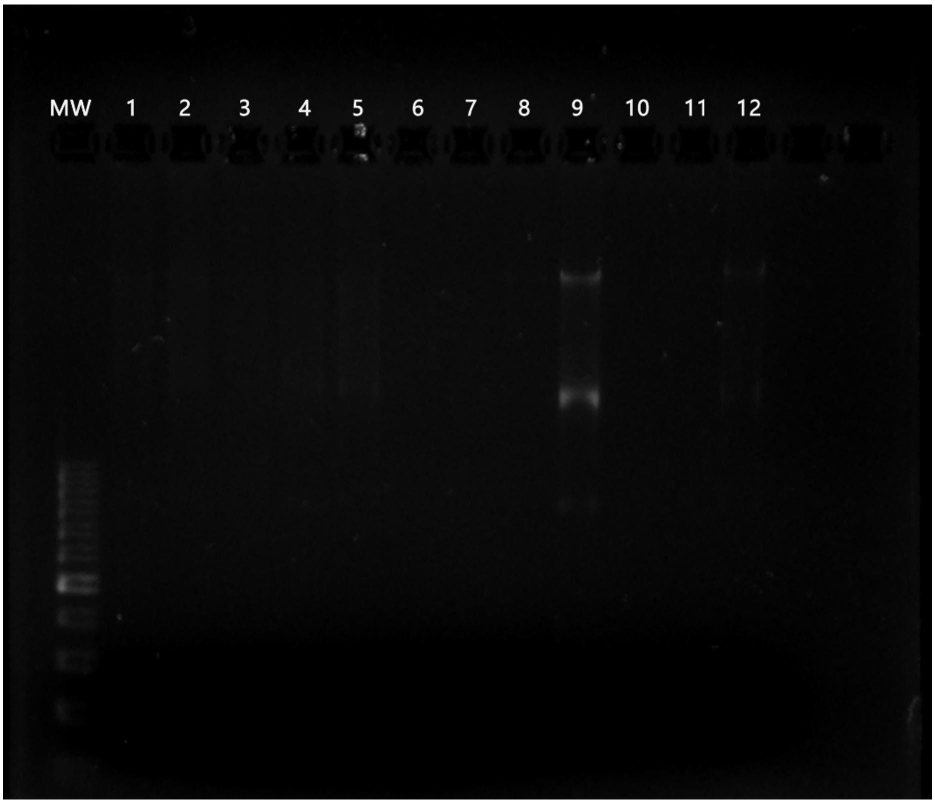

S7 Fig. Agarose gel electrophoresis of testis samples preserved in RNAlater® for 30 days at RT or 4 ºC. 1-6: testis samples stored at RT; 7-12: testis samples stored at 4 ºC; MW: molecular weight marker.

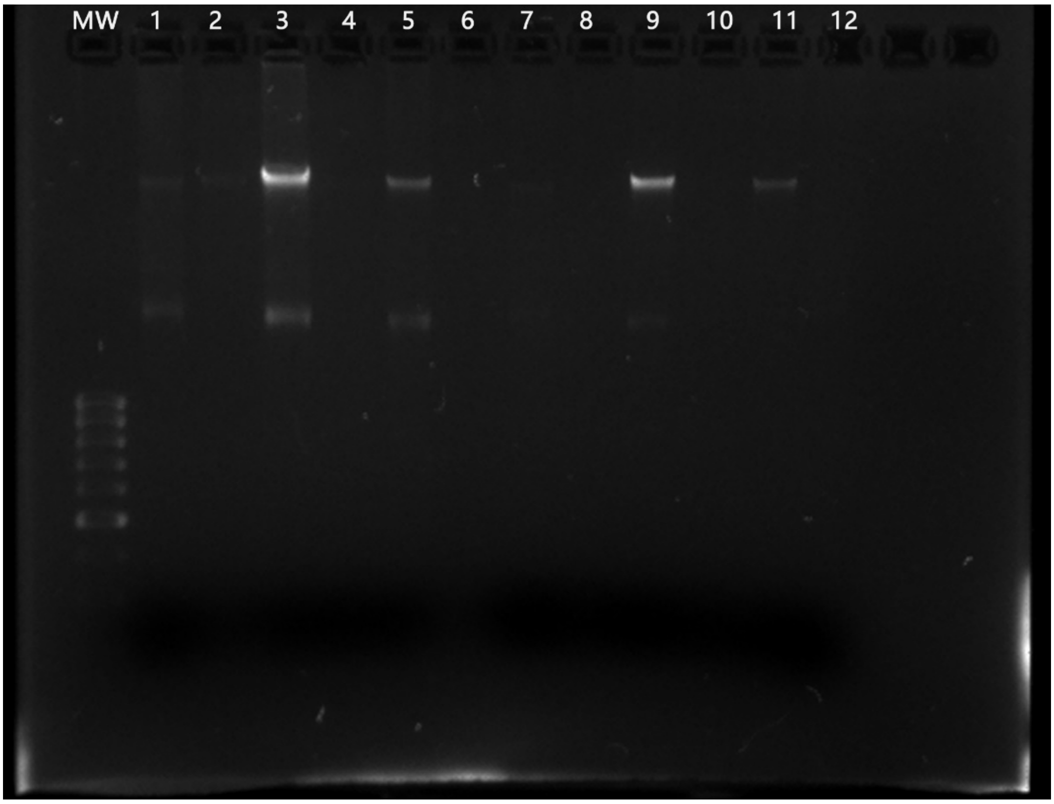

S8 Fig. Agarose gel electrophoresis of testis samples preserved in RNAlater® for 30 days at -20 ºC or -80 ºC. 1-6: testis samples stored at -20 ºC; 7-12: testis samples stored at -80 ºC; MW: molecular weight marker.

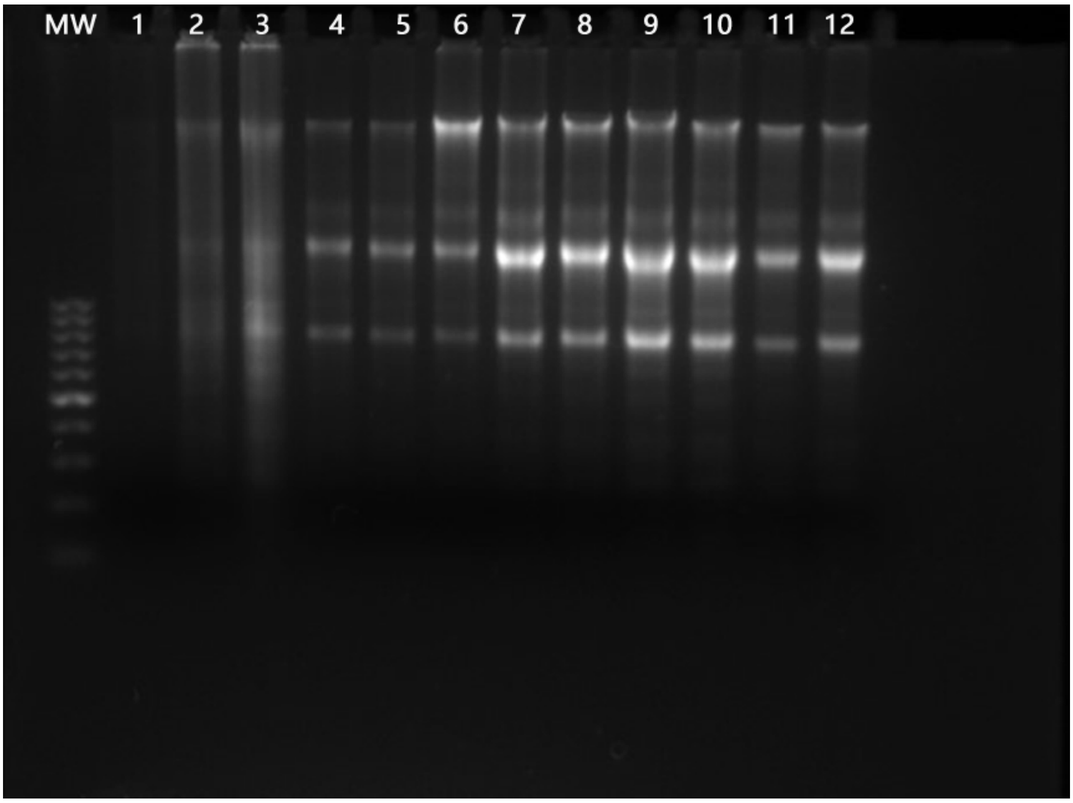

S9 Fig. Agarose gel electrophoresis of testis samples preserved in RNAlater® for 90 days at different temperatures. 1, 2 and 3: testis samples stored at RT; 4,5 and 6: testis samples stored at 4 ºC; 7, 8 and 9: testis samples stored at -20 ºC; 10, 11 and 12: testis samples stored at -80 ºC; MW: molecular weight marker.

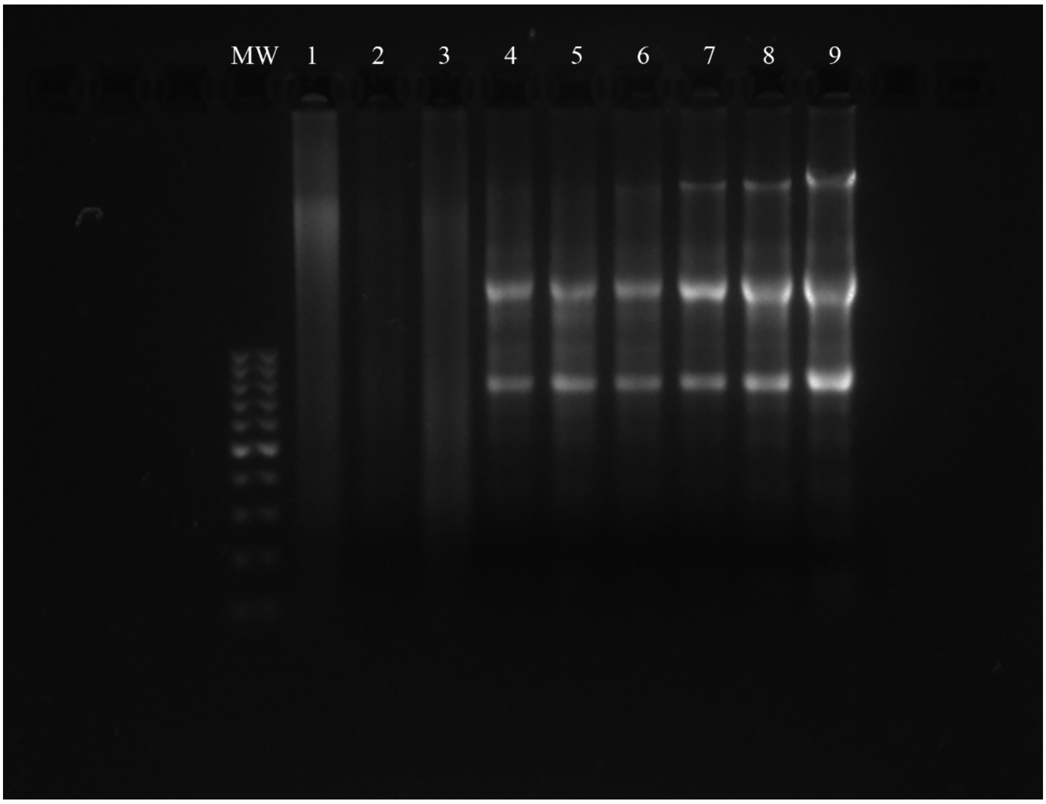

S10 Fig. Agarose gel electrophoresis of testis samples preserved in RNAlater® for 365 days at different temperatures. 1, 2 and 3: testis samples stored at RT; 4,5 and 6: testis samples stored at 4 ºC; 7, 8 and 9: testis samples stored at -20 ºC; MW: molecular weight marker.

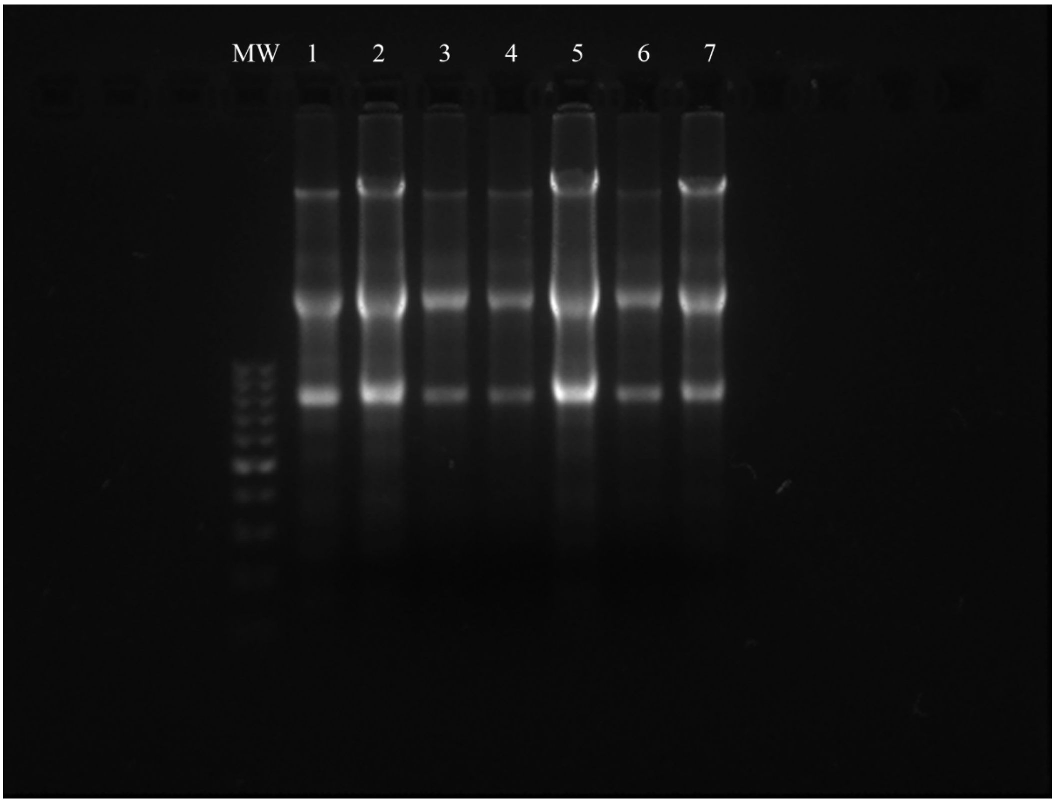

S11 Fig. Agarose gel electrophoresis of testis samples stored for 365 days at different storage conditions. 1 and 2: testis samples snap-frozen and then stored at -80 ºC; 3 and 4: testis samples snap-frozen and then stored in liquid nitrogen (LN_2); 5, 6 and 7: testis samples preserved in RNAlater® and stored at -80 ºC; MW: molecular weight marker.
